# *Aconitum carmichaelii*: a clinical-regulatory synthesis of traditional use, toxicological evidence, and global safety governance

**DOI:** 10.3389/fphar.2026.1830589

**Published:** 2026-06-22

**Authors:** Ru Ya Tan, Pin Jun Ooi, Xiangyang Xu, Xu Wei, Xu Wen, Qiyue Qiu, Mun Fei Yam

**Affiliations:** 1 School of Pharmaceutical Sciences, Universiti Sains Malaysia, Minden, Pulau Pinang, Malaysia; 2 College of Pharmacy, Fujian University of Traditional Chinese Medicine, Fuzhou, Fujian, China

**Keywords:** aconitine, *Aconitum carmichaelii*, clinical toxicology, evidence hierarchy, Fuzi, quality control, regulatory harmonisation, risk-benefit assessment

## Abstract

*Aconitum carmichaelii* Debeaux (Ranunculaceae), commonly used as Aconiti Lateralis Radix Praeparata (Fuzi), is a clinically important but highly toxic herb in traditional Chinese medicine. Despite long-standing use for cardiovascular, rheumatologic, gastrointestinal, and pain-related conditions, its application is constrained by a narrow therapeutic index, variable processing practices, and recurrent reports of poisoning. Given the substantial number of existing reviews on aconite, this article is deliberately positioned as a clinical-regulatory synthesis rather than another general phytochemical or mechanistic overview. It complements recent integrative-toxicology reviews by linking phytochemistry, pharmacodynamics, toxicodynamics, clinical poisoning patterns, evidence hierarchy, processing variability, analytical quality control, and international regulatory governance. A structured literature search, informed by PRISMA 2020 reporting principles, was conducted across PubMed, CNKI, Web of Science, Scopus, and ScienceDirect for English- and Chinese-language publications available up to 31 December 2025, supplemented by pharmacopoeial and regulatory documents. Evidence was synthesised narratively because of heterogeneity in study design, model systems, preparations, and outcome measures. Diterpenoid alkaloids, especially diester diterpenoid alkaloids, are central to both bioactivity and toxicity. Preclinical studies suggest cardiotonic, anti-inflammatory, analgesic, immunomodulatory, and anti-tumour effects, but many claims remain based on *in vitro* or animal models, isolated compounds, or multi-herb formulas in which Fuzi-specific effects cannot be separated. In contrast, clinical poisoning data consistently link excessive or inadequately processed exposure to cardiotoxicity and neurotoxicity through ion-channel disruption, calcium overload, mitochondrial dysfunction, oxidative stress, and apoptosis. The strongest human evidence relates to standardised proprietary formulations tested in larger controlled trials, whereas most decoction-based reports remain small, adjunctive, or methodologically limited. Overall, the novel contribution of this review is a practice-oriented risk-benefit framework that connects mechanistic plausibility with real-world clinical toxicology, quality-control checkpoints, and global safety governance.

## 1 Introduction

*Aconitum carmichaelii* Debeaux (Ranunculaceae) is a commonly used traditional herb in Asian countries such as China, India, Japan, and Korea (Zhou et al., 2015). Although this plant is native mainly to China, particularly the Sichuan Province, it can be found in other countries like Vietnam, the Netherlands, Japan, and the United States of America (Fu et al., 2022). According to the Pharmacopoeia of the People’s Republic of China (2025) (ChP), the medicinal materials derived from *Aconitum carmichaelii* Debeaux include Aconiti Lateralis Radix Praeparata (Fuzi), the processed lateral root (daughter root), which is the most used medicinal part, and Aconiti Radix (Chuanwu), the main (mother) root. Nevertheless, due to the high toxicity of raw aconite roots, only processed forms are permitted for medicinal use in most pharmacopoeias. The recognized processed forms include Yanfuzi (salt-processed), Heishunpian (black sliced aconite), Baifupian (white sliced aconite), and Paofuzi (processed/boiled aconite) (Chinese Pharmacopoeia Commission, 2025).

The historical use of Aconitum species was first documented in Shennong’s Materia Medica, the earliest known Chinese herbal text (Chan et al., 2021). Owing to their hot and dry nature, they can resist cold environments and thus have been used for conditions like cold limbs, painful knees, difficulties in walking, poor circulation, and spasms (Nyirimigabo et al., 2015; Tai et al., 2015). Traditionally, residents of rural villages in the Qinling Range of the Shaanxi Province in China consumed *Aconitum carmichaelii* before winter, believing it could help generate warmth and enhance their energy for daily work. India also used Aconitum species to treat fever, inflammation, vomiting, and diarrhoea (Jaiswal et al., 2014).

Nowadays, Fuzi is often consumed with other herbs to treat shock due to acute myocardial infarction, hypotension, coronary heart disease, and chronic heart failure (Zhao et al., 2012). Meanwhile, Fuzi is also widely used to treat a wide range of diseases, including rheumatoid arthritis, tumours, diarrhoea, gastroenteritis, oedema, and some endocrine disorders like irregular menstruation and dysmenorrhea (Hu et al., 2023; Liu et al., 2025; Singhuber et al., 2009; Wu et al., 2018; Zhou et al., 2015). This is because *Aconitum carmichaelii* has anti-inflammatory, analgesic, and antitumor activities, attributable to alkaloids as the major bioactive compounds (Zong et al., 2019). Moreover, polysaccharides and phenolic compounds have exhibited anti-inflammatory, immunomodulatory, anti-oxidative, anti-tumour, and cardioprotective effects as well (Fu et al., 2022).

Undeniably, *Aconitum carmichaelii* has several therapeutic benefits, but its improper use can cause cardiotoxicity and neurotoxicity due to the alkaloid content. Shennong’s Materia Medica classified it as a “lower class” drug and indicated it as “very poisonous” which must be handled with extra caution (Liang et al., 2025). In fact, about 5,000 aconite poisoning incidents have been reported in areas that practice herbal medicines, especially in China and Hong Kong (Chan, 2015; Yang M. et al., 2018). The common poisoning symptoms are dizziness, palpitation, nausea, vomiting, arrhythmia, shock, and coma (Singhuber et al., 2009).

Notably, traditional detoxification processes like boiling, steaming, and salt processing, as well as herbal combination therapy, have been shown to reduce the herb’s toxicity while preserving therapeutic activity (Chan et al., 2021). At the same time, the use of recent analytical methods such as high-performance liquid chromatography (HPLC) allows for the detection and quantification of alkaloids (Zong et al., 2019). Although both conventional and modern approaches have been adopted to mitigate its toxicity, a standard guideline is still lacking to ensure its safe and effective use, as the regulation of *Aconitum carmichaelii* in current pharmacopoeias still varies widely among countries, restricting its application to the current clinical practice.

Overall, *Aconitum carmichaelii* represents a clinically relevant but high-risk medicinal herb whose interpretation requires simultaneous consideration of traditional use, pharmacological plausibility, human safety data, and regulatory control. The available evidence is scattered across phytochemistry, preclinical pharmacology, toxicology, case reports, clinical studies, and pharmacopoeial standards, and many publications describe efficacy without sufficiently discussing dose relevance, preparation type, evidence quality, or safety margins. This review therefore aims to integrate these domains within a clinical-regulatory risk-benefit framework, explicitly distinguishing mechanistic promise from clinically supported use and clarifying where pharmacodynamic benefit and toxicodynamic injury arise from shared alkaloid-mediated pathways (Feng et al., 2021; Fu et al., 2022).

### 1.1 Novelty and added value relative to recent reviews

The novelty of this review should be understood in relation to the existing review landscape. Multiple reviews and meta-analyses have already summarised aconite chemistry, pharmacology, toxicity, detoxification and selected clinical uses; therefore, the added value of the present manuscript cannot rest on repeating those domains descriptively. A particularly relevant comparator is the recent review by Li et al. (2025), which introduces an integrative toxicology framework to bridge the traditional toxicity-efficacy concept with modern toxicological mechanisms (Li et al., 2025). That article provides an important conceptual and mechanistic perspective, especially on TCM processing and compatibility, but it does not primarily function as a clinical-regulatory synthesis.

In contrast, the present review is deliberately practice-oriented. It integrates mechanistic toxicology with real-world poisoning patterns, compares the strength of human clinical evidence across proprietary formulations and classical decoctions, evaluates processing and analytical quality-control challenges, and summarises international regulatory approaches across China, Taiwan, Japan, Korea, India, Australia, Southeast Asia, North America and Europe. This clinical-toxicological and regulatory framing is intended to support safer interpretation by clinicians, pharmacologists, toxicologists, product developers and regulators, and to complement rather than duplicate prior mechanistic or TCM-theory-based reviews.

## 2 Methods

### 2.1 Review design and reporting framework

This article was prepared as a structured review with a transparent literature identification and screening process informed by the PRISMA 2020 statement (Page et al., 2021). Because the included evidence comprised *in vitro* studies, animal experiments, clinical studies, case reports, pharmacopoeial documents, and regulatory sources, the findings were synthesized narratively rather than pooled in a meta-analysis. The accepted scientific name of the species reviewed in this manuscript was verified as *Aconitum carmichaelii* Debeaux (family Ranunculaceae) using Plants of the World Online (Plants of the World Online, 2026).

### 2.2 Information sources and search strategy

Searches were conducted from database inception to 31 December 2025 in PubMed, CNKI, Web of Science, Scopus and ScienceDirect. Additional hand-searching of reference lists and relevant pharmacopoeial or regulatory documents was undertaken to capture jurisdiction-specific safety standards and grey literature that might not be indexed consistently across bibliographic databases.

The search strategy was adapted to each database by combining botanical names, common names, constituent names, and topic-specific terms. The core search string used combinations of free-text terms such as “*Aconitum carmichaelii*” OR Fuzi OR “Aconiti Lateralis Radix Praeparata” OR aconitine with phytochemistry OR pharmacology OR toxicology OR toxicity OR poisoning OR safety OR “case report” OR regulation OR pharmacopoeia. Targeted supplementary searches were also performed for specific preparations and clinically relevant topics, including Shenfu Injection, Qili-qiangxin capsules, cardiotoxicity, and neurotoxicity.

### 2.3 Eligibility criteria

Sources were eligible for qualitative synthesis when they were original research articles, clinical studies, case reports, toxicological investigations, pharmacological or phytochemical studies, or official pharmacopoeial and regulatory documents published in English or Chinese. Relevant review articles were consulted for citation chaining and contextual background, but primary data and original source documents were preferentially extracted whenever available. Records were excluded when botanical identity was unclear, when the report focused on unrelated *Aconitum* species without extractable data for *Aconitum carmichaelii*, when the full text could not be evaluated, or when the publication did not provide primary or regulatory information relevant to the objectives of this review.

### 2.4 Study selection and screening process

**FIGURE 1:**
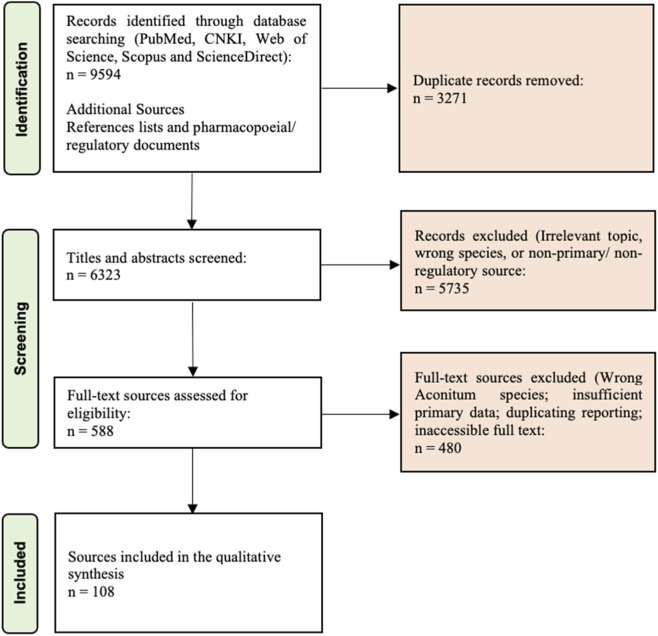
PRISMA-style flow diagram of literature identification, screening, eligibility assessment, and inclusion.

All retrieved records were first examined for duplication and obvious irrelevance. Title and abstract screening was then performed using the predefined eligibility criteria, followed by full-text evaluation of potentially relevant sources. Reasons for exclusion at full-text stage included wrong species, insufficient relevance to the predefined review domains, lack of usable primary or regulatory information, duplicate reporting, and inaccessible full text. The overall selection pathway is summarized in the PRISMA-style flow diagram shown in Figure 1.

In total, n = 9,594 records were identified from databases. After removal of n = 3,271 duplicates, n = 6,323 records underwent title and abstract screening, n = 588 full-text sources were assessed for eligibility, and n = 108 sources were included in the final qualitative synthesis.

### 2.5 Data extraction and synthesis

For each included source, information was extracted on publication type, study model, Fuzi or aconite preparation, principal therapeutic or toxicological findings, safety-related measures, and regulatory context. During synthesis, the evidence was also interpreted according to its likely clinical relevance, with preclinical mechanistic findings considered separately from human data whenever direct extrapolation would be inappropriate. The evidence was then organised into thematic domains, namely, phytochemistry, pharmacodynamics, toxicodynamics, clinical applications and poisoning incidents, safety considerations, and regulatory challenges. This structure supported a narrative synthesis appropriate to the heterogeneity of the included materials.

### 2.6 Evidence appraisal and risk-benefit synthesis

Because the evidence base included *in vitro* experiments, animal models, formula-level studies, randomised and non-randomised clinical studies, case reports, pharmacopoeial monographs, and regulatory documents, a single pooled certainty estimate was not appropriate. Instead, evidence was appraised narratively according to study design, human relevance, preparation standardisation, dose or exposure reporting, safety monitoring, outcome type, and whether the contribution of Fuzi could be separated from other ingredients. Human randomised trials of standardised proprietary formulations were considered more clinically informative than isolated cell experiments, animal studies, uncontrolled case series, or mechanistic network-pharmacology predictions. For safety conclusions, clinical poisoning reports and regulatory documents were interpreted alongside mechanistic toxicology to connect real-world exposure patterns with observed cardiovascular and neurological outcomes.

### 2.7 Positioning relative to previous reviews

During synthesis, recently published reviews on Aconitum/Fuzi were also considered to identify where the present manuscript could add value rather than duplicate existing work. Special attention was given to Li et al. (2025), a recent Frontiers in Pharmacology review centred on integrative toxicology (Li et al., 2025). The present review was therefore organised to emphasise domains that remain less developed in prior reviews: evidence hierarchy for human use, case-based clinical toxicology, proprietary preparation data, international regulatory comparison, finished-product quality control, hidden or unstable alkaloid concerns, and global harmonisation needs.

## 3 Phytochemistry

The chemical composition of *Aconitum carmichaelii* is highly complex, with diterpenoid alkaloids being the primary bioactive and toxicologically relevant constituents. Non-alkaloidal components, including polysaccharides, flavonoids, saponins, carbohydrates, fatty acids, ceramides, and glycosides, have also been reported and may contribute supportive pharmacological effects (Fu et al., 2022; Zhou et al., 2015).

### 3.1 Alkaloids

Among the 122 chemical constituents identified in *Aconitum carmichaelii*, 105 alkaloids are reported, making them the primary components of the plant with C19-diterpenoid alkaloids being the most abundant ones. The alkaloids can be further categorized according to the number of ester bonds into diester-diterpenoid alkaloids (DDAs), monoester-diterpenoid alkaloids (MDAs) and aminol-diterpenoid alkaloids (ADAs), which could act as quality marker compounds for the chemical analysis of Fuzi (Liang et al., 2025; Wu et al., 2018; Zhou et al., 2015).

**FIGURE 2:**
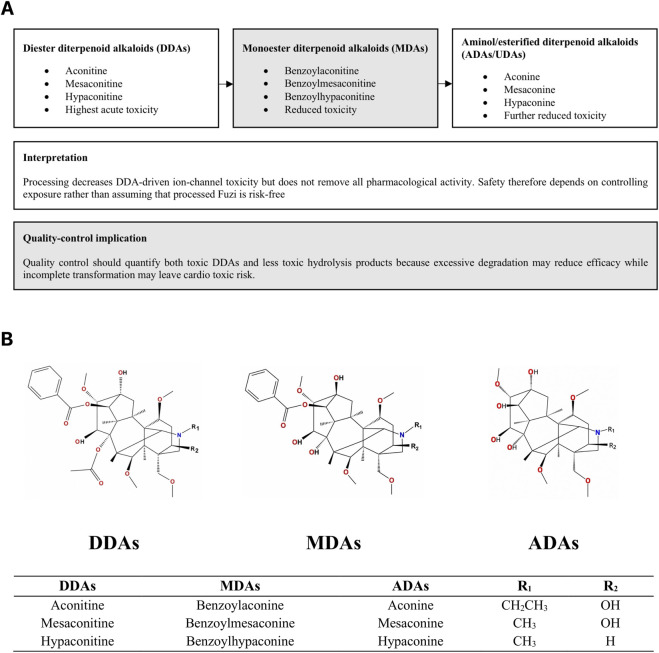
**(A)** Schematic transformation and **(B)** chemical structure of toxic diester diterpenoid alkaloids and their less toxic hydrolysis products during Fuzi processing.

Most C19-diterpenoid alkaloids follow the prototypical aconitine scaffold, and their safety profile is strongly influenced by ester substitution and processing-related hydrolysis, as summarised schematically in Figure 2 (Zhao et al., 2024; Zhou et al., 2015). DDAs containing C8-acetyl and C14-benzoyl ester groups are generally the most toxic (Ameri, 1998). Aconitine is a DDA with both an acetyl group at C8 and a benzoyl group at C14 of a complex hexacyclic framework with 15 chiral centres that contribute to its biological activity and toxicity (Gao et al., 2022; Zhao et al., 2024). Upon heating or hydrolysis, DDAs such as aconitine, mesaconitine, and hypaconitine can be transformed first into MDAs such as benzoylaconine, benzoylmesaconine, and benzoylhypaconine, and subsequently into less esterified alkaloids such as aconine, mesaconine, and hypaconine (Mizugaki et al., 1998; Wada et al., 2005). Therefore, substitution or hydrolysis at these ester positions reduces toxicity and modifies pharmacological activity rather than simply removing all bioactivity (Gao et al., 2022; Wang Y. J. et al., 2021).

*Aconitum carmichaelii* contains high levels of C19-diterpene alkaloids that provide it with analgesic activity, myocardial protection, and moderate anti-cancer effects. Other than that, C20-diterpenoid alkaloids that show analgesic and anti-cancer effects can also be detected in *Aconitum carmichaelii* (Liang et al., 2025). ADAs are also present and provide additional effects for treating the neurological disorders, reducing inflammation, and facilitating wound healing (Tao et al., 2013).

Phenylpropanoids, fatty acids, organic acids and polysaccharides are non-alkaloid components found in *Aconitum carmichaelii*. They can be utilised to treat inflammation and cardiovascular diseases (Liang et al., 2025). Meanwhile, polysaccharides can give additional effects such as immunomodulatory, anti-diabetic, anti-tumour, anti-oxidative, cholesterol-lowering and anti-HIV-1 reverse transcriptase activities (Fu et al., 2022).

### 3.2 Polysaccharides

Polysaccharides make up a substantial portion of the dry weight of *Aconitum carmichaelii,* with reported content ranging from 3.3% to 33.5% in Fuzi and 20%–30% in Chuanwu (Lv et al., 2011; Zhao X. S. et al., 2009). However, the content is affected by factors including geographical origin, harvest time, storage conditions, and plant hormone levels (Shu and Hou, 2008; Shu et al., 2009; Yue, 2015; Zhao X. et al., 2009). Starch is the major polysaccharide, constituting 73.9% of total carbohydrates in Fuzi and 55.0% in Chuanwu (Gao et al., 2010). Additionally, several water-soluble glucans (e.g., aconitan A, FPS-1, FI) and heteropolysaccharides containing sugars like rhamnose, arabinose, galactose, glucose, mannose, xylose, and uronic acids have been isolated (Fu et al., 2022). Processing can decrease the polysaccharide content as a result of carbohydrate loss following boiling or steaming (Fu et al., 2022).

### 3.3 Phenolic compounds

To date, 39 phenolic compounds have been identified from the roots, which are broadly classified into flavonoids, lignans, phenylpropanoids, benzoic acid derivatives, and other simple phenolics (Fu et al., 2022). Notably, flavonoids are regarded as the chemotaxonomic biomarkers within the genus other than alkaloids (Yin et al., 2019). Although present at lower levels compared to alkaloids, these compounds contribute to the chemical fingerprint of *Aconitum carmichaelii* and may have supporting roles in its pharmacological activities (Fu et al., 2022).

## 4 Pharmacodynamics and toxicodynamics

Systems pharmacology studies suggest that some of the compounds and targets contributing to the therapeutic activity of *Aconitum carmichaelii* also underlie its toxicity (Feng et al., 2021). Accordingly, the pharmacological profile of *Aconitum carmichaelii* is better understood as an efficacy-toxicity continuum rather than as two independent domains. The same diterpenoid alkaloids and molecular targets may support therapeutic responses when exposure is controlled, yet produce overt toxicity when concentration, duration, or absorption exceeds the narrow therapeutic window. At the same time, this framework remains largely conceptual and preclinical; it helps explain mechanistic overlap, but it does not by itself establish clinical efficacy in humans. This overlap appears strongest for diterpenoid alkaloids, especially DDAs and their less toxic hydrolysis products, whereas non-alkaloid constituents such as polysaccharides may contribute supportive effects with comparatively less evidence for acute poisoning (Feng et al., 2021; Fu et al., 2022).

### 4.1 Quality and limitations of current evidence

The current evidence base is extensive but uneven. Pharmacodynamic claims are largely supported by *in vitro* experiments, animal models, network-pharmacology analyses, or studies of multi-herb formulas; therefore, their relevance to human exposure after clinically processed Fuzi is not always clear. Many studies do not report alkaloid exposure in a way that can be compared with pharmacopoeial limits or clinical dosage, and some mechanistic findings differ between models, such as rodent cardiomyocytes and human induced pluripotent stem cell-derived cardiomyocytes. Clinical studies are also heterogeneous: Fuzi is usually embedded in a formula, proprietary product, or adjunctive intervention, so the specific contribution of Fuzi cannot be isolated confidently. By contrast, the toxicology evidence is more convergent because mechanistic studies, poisoning cases, and regulatory restrictions consistently identify DDA-rich exposure as a major cause of arrhythmia, neurotoxicity, and cardiovascular collapse. For this reason, the review interprets efficacy claims cautiously while treating safety signals as clinically actionable.

### 4.2 Pharmacodynamics

The pharmacodynamic activities attributed to *Aconitum carmichaelii* are mainly linked to diterpenoid alkaloids and polysaccharides. Experimental studies describe a broad range of biological effects, including cardiovascular support, anti-inflammatory activity, analgesia, immunomodulation, and anti-tumour actions. These effects are generally mediated through modulation of ion channels, inflammatory signalling pathways, and cellular metabolic processes. However, most of this literature comes from *in vitro* systems, animal models, or formula-level experiments, so these findings should be interpreted as mechanistic or hypothesis-generating rather than as direct proof of clinical benefit in humans.

#### 4.2.1 Cardiovascular effects

The cardiovascular activity of *Aconitum carmichaelii* is among its most extensively studied pharmacological properties. Diterpenoid alkaloids, particularly aconitine and related compounds, can exert positive inotropic effects in experimental systems through modulation of voltage-gated sodium channels (Bi et al., 2020). Aconitine prolongs sodium-channel activation, resulting in sustained sodium influx that subsequently alters the activity of the Na^+^-Ca^2+^ exchanger (NCX). This process elevates intracellular calcium levels and may enhance cardiomyocyte contractility under controlled conditions (Chelko et al., 2019). At low concentrations, this mechanism may help explain cardiotonic signals observed in experimental models, particularly under disease-like conditions such as heart failure (Kimura et al., 1994; Liu et al., 2012). In addition, benzoylaconine, mesaconine, and Fuzi polysaccharides have been reported to influence anti-arrhythmic signalling, mitochondrial homeostasis, antioxidant responses, or autophagy-related pathways (Liao et al., 2013; Nyirimigabo et al., 2015; Yang et al., 2021; Zhang et al., 2022; Zhou et al., 2023). Nevertheless, the clinical relevance of these findings remains uncertain because most studies involve isolated cells, rodents, or experimental exposure conditions that do not fully reflect processed clinical Fuzi use. Moreover, the strongest human data in this area relate to multi-herb formulations or proprietary products rather than isolated aconite alkaloids. Thus, these results are better interpreted as translational leads than as confirmed cardiotonic efficacy in patients. In parallel, the same sodium-channel and calcium-handling pathways that may underlie experimental benefit can drive delayed afterdepolarisations, calcium overload, and arrhythmia when exposure is higher or more rapidly absorbed (Sun et al., 2014; Wu et al., 2017; Zhang et al., 2022).

#### 4.2.2 Anti-inflammatory effects

Preclinical studies also suggest anti-inflammatory activity, primarily linked to alkaloids and bioactive constituents present in traditional herbal formulations. At the molecular level, these effects have been associated with suppression of MAPK phosphorylation and inhibition of transcription factors such as NF-kappaB, STAT3, and JAK2 (Huang et al., 2022). Fuzi-derived preparations have also been reported to reduce pro-inflammatory cytokines and modulate immune responses in cell and animal models. For example, Fuzi-Lizhong-related studies describe changes in gut microbiota composition and reductions in mediators such as TNF-alpha, IL-1beta, IFN-gamma, and IL-6 (Zhang et al., 2024; Zhen et al., 2021). In addition, Fuzi-Lizhong decoction has shown experimental effects on metabolic and inflammatory pathways, including TLR4/MyD88/TRAF6/NF-kappaB signalling, in models of metabolic dysfunction (Yang et al., 2022). These findings are biologically interesting, but they remain largely preclinical and frequently reflect formula-level effects, so they should not be read as definitive evidence of anti-inflammatory efficacy of Fuzi in patients.

#### 4.2.3 Analgesic effects

Analgesic activity has likewise been described mainly in animal models, where aconitine reduced cold and mechanical allodynia in mice with cancer-induced bone pain at doses as low as 0.05 mg/kg (Jin et al., 2023). Proposed mechanisms involve modulation of nociceptive ion channels, including TRPA1 and TRPV1, and possible interaction with cannabinoid-related signalling pathways (Jin et al., 2023; Zhao et al., 2024). These data support mechanistic plausibility for antinociceptive effects, but they do not yet establish clinical analgesic efficacy in humans. This distinction is especially important because the same excitable-cell pathways implicated in experimental pain relief are also linked to neurotoxicity when exposure becomes excessive, sustained, or insufficiently controlled by processing (Jin et al., 2023; Peng et al., 2009; Zhao et al., 2024).

#### 4.2.4 Immunomodulatory and anti-tumour effects

Immunomodulatory and anti-tumour effects have also been reported, but the evidence here is even more clearly preclinical. FPS have been described as enhancing immune-cell activity and resistance to immunosuppression in experimental systems (Fu et al., 2022). Likewise, several Fuzi-derived compounds have shown pro-apoptotic or anti-proliferative effects in tumour cell lines or animal models (Du et al., 2013; Li et al., 2017; Xiang et al., 2023). These findings are useful for generating mechanistic hypotheses, yet they should not be interpreted as evidence that *Aconitum carmichaelii* or its isolated alkaloids are clinically established anti-cancer therapies. In particular, tumour-cell apoptosis *in vitro* does not necessarily translate into a favourable therapeutic index in humans, especially for a herb with well-recognised cardiotoxic and neurotoxic liabilities. Any discussion of anti-tumour potential therefore needs to remain cautious and clearly framed as preliminary rather than clinically proven.

Taken together, the pharmacodynamic literature is dominated by *in vitro* work, animal models, and formulation-level experiments. These studies are valuable for understanding mechanisms and generating clinically relevant questions, but they do not on their own confirm efficacy in patients. At present, the most direct human relevance comes from selected Fuzi-containing formulas and proprietary preparations, and even in those settings, attribution of benefit specifically to Fuzi is often difficult (Cheang et al., 2024; Ji et al., 2023; Song et al., 2012; Zhang et al., 2020).

### 4.3 Toxicodynamics

Despite its therapeutic potential, *Aconitum carmichaelii* is well known for its significant toxicity, mainly due to the presence of DDAs (aconitine, mesaconitine, and hypaconitine) being the most toxic constituents, thereby restricting its clinical uses and approval (Liang et al., 2025; Nyirimigabo et al., 2015). The LD50 of aconitine for mice is 1.8 mg/kg through oral administration while a single dose at 2–6 mg is reported to be fatal in humans (Wang Y. et al., 2006). These compounds exert toxic effects mainly through disruption of ion channel function, mitochondrial dysfunction, oxidative stress, and apoptosis signaling pathways. Remarkably, the toxicity of hydrolyzed aconitine, benzoylaconine, a C19 MDA, is decreased by a factor of 38 in mice (Zhang et al., 2017). Although hydrolysis can reduce the toxicity, overdose and overabsorption can still result in poisoning due to its cardiotoxic and neurotoxic effects. Thus, toxicodynamics can be interpreted as an extension of the same alkaloid-driven bioactivity beyond the safe therapeutic window rather than as a wholly separate mechanistic process. Importantly, this shared framework does not imply that all beneficial constituents are equally hazardous; rather, the most direct efficacy-toxicity coupling is seen with DDA-rich alkaloid fractions, while processed preparations and non-alkaloid components may partially dissociate efficacy from acute poisoning risk (Fu et al., 2022; Ji et al., 2023).

#### 4.3.1 Cardiotoxicity

Cardiotoxicity represents the most serious adverse effect associated with aconite poisoning. The toxic effects are primarily mediated through the interaction of aconitine with voltage-gated sodium channels in cardiomyocytes. Aconitine binds to the specific neurotoxin binding site 2 of the α-subunit of these channels, locking them in an open state and preventing their normal inactivation, resulting in persistent sodium influx and prolonged membrane depolarisation. This disruption of ion homeostasis subsequently leads to intracellular calcium overload through activation of the NCX. Elevated intracellular calcium levels can trigger abnormal electrical activity and increase myocardial excitability, ultimately causing arrhythmias (Ameri, 1998; Catterall, 1988). Notably, species-dependent differences in the electrophysiological response to aconitine have been observed. While aconitine increased the L-type Ca^2+^ current (ICa,L) density and prolonged action potential duration in rat ventricular myocytes (Zhou et al., 2013), studies using human induced pluripotent stem cells-derived cardiomyocytes (hiPSC-CMs) reported inhibition of L-type Ca^2+^ channels and action potential duration shortening (Wu et al., 2017). These discrepancies may reflect interspecies differences in ion channel expression, calcium handling mechanism, and the immature phenotype of hiPSC-CMs. Nevertheless, both models consistently demonstrated triggered activities and delayed afterdepolarizations, suggesting that intracellular Ca^2+^ dysregulation remains a central arrhythmogenic mechanism. In addition to electrophysiological disturbances, aconitine-induced calcium overload may also impair mitochondrial function and induce cardiomyocyte apoptosis, further contributing to cardiac injury (Peng et al., 2020; Sun et al., 2014; Zhao et al., 2024). Evidence from *in vivo* rodent studies and meta-analyses also highlights that aconitine-induced arrhythmias and myocardial damage are consistently associated with ROS generation and inflammasome activation (Jiang et al., 2022). These mechanisms explain the frequent occurrence of arrhythmias and cardiovascular collapse observed in severe aconite poisoning cases.

#### 4.3.2 Neurotoxicity

Neurotoxicity is another prominent toxic effect of aconite alkaloids due to their potent actions on neuronal sodium channels. Persistent activation of voltage-gated sodium channels in neurons results in prolonged depolarization and abnormal neuronal excitability, which can disrupt normal neurotransmission (Muroi et al., 1990; Peng et al., 2009). Clinically, this manifests as neurological symptoms such as paresthesia, numbness of the perioral region and extremities, dizziness, and muscle weakness following exposure to aconite-containing products. At the cellular level, studies using neuronal cell models have shown that aconitine can induce mitochondrial dysfunction, impair energy metabolism, and promote oxidative stress (Pang et al., 2023; Zhao et al., 2010). Disruption of mitochondrial dynamics and inhibition of AMP-activated protein kinase signaling pathways have also been reported, leading to reduced ATP production and increased susceptibility to neuronal injury (Wang et al., 2022; Yang et al., 2021). In addition, prolonged ionic imbalance may trigger downstream pathways associated with apoptosis and cellular damage in neuronal cells (Jiang et al., 2023). These combined mechanisms contribute to the neurotoxic profile of aconite alkaloids and highlight their potential to cause both acute neurological symptoms and cellular toxicity.

### 4.4 Integrated pharmacodynamic-toxicodynamic framework

Taken together, the reviewed evidence supports an integrated pharmacodynamic-toxicodynamic framework for *Aconitum carmichaelii*. In mechanistic terms, therapeutic efficacy and toxicity are not produced by entirely different chemical systems; instead, they arise from overlapping alkaloid-mediated actions that diverge according to dose, exposure rate, processing status, formulation context, and patient susceptibility. For example, limited modulation of voltage-gated sodium channels and downstream calcium handling may contribute to positive inotropy or analgesic signalling, whereas persistent channel opening drives membrane depolarisation, calcium overload, electrical instability, and tissue injury.

A similar overlap is seen in mitochondrial and inflammatory pathways. Under controlled conditions, some aconite-derived compounds or processed preparations have been associated with improved mitochondrial homeostasis, adaptive mitophagy, and suppression of inflammatory mediators. However, when exposure becomes excessive or inadequately processed DDAs predominate, the same cellular systems shift toward oxidative stress, inflammasome activation, mitochondrial dysfunction, and apoptosis. In this sense, toxicodynamic injury can be viewed as a quantitative and contextual escalation of pharmacological activity rather than a wholly unrelated phenomenon (Feng et al., 2021; Zhang et al., 2022).

**FIGURE 3:**
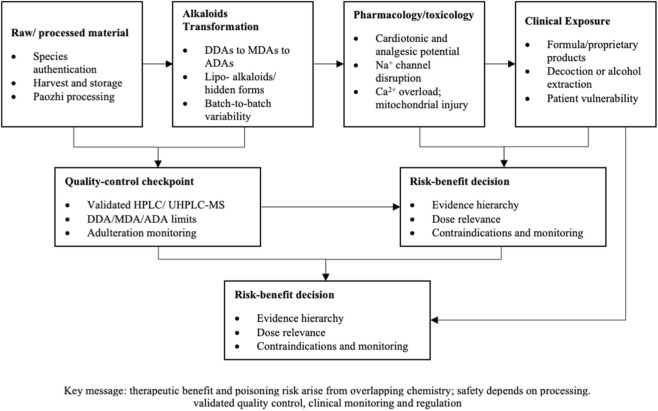
Conceptual model linking raw material control, alkaloid transformation, pharmacology-toxicology overlap, clinical exposure, quality-control checkpoints, and regulatory governance for safer clinical use of Fuzi.

This interpretation also helps explain the central role of processing and compatibility in Fuzi-based therapy. Hydrolysis of DDAs into less toxic MDAs does not simply eliminate activity; rather, it rebalances the efficacy-toxicity relationship by reducing peak ion-channel disruption while retaining part of the desired pharmacological effect. Likewise, multi-herb formulations may widen the therapeutic window by modifying absorption, metabolism, oxidative stress, or downstream tissue vulnerability. The conceptual relationships among raw material control, alkaloid transformation, pharmacological and toxicological pathways, clinical exposure, and regulatory governance are summarised in Figure 3. Operationally, the integrated model can be reduced to four mechanistic bridges-ion-channel regulation, calcium/mitochondrial homeostasis, inflammatory signalling, and context-dependent apoptosis-whose clinical direction is gated by dose, processing, formulation, route of exposure, and tissue susceptibility (Chan et al., 2021; Feng et al., 2021).

## 5 Clinical evidence and poisoning cases

It is important to examine the use of *Aconitum carmichaelii* in real-world settings to better understand its dual nature. Clinical reports provide some translational context, but they do not validate all preclinical pharmacological claims, especially when Fuzi is administered as part of multi-herb formulas or proprietary products. Accordingly, the case literature is most useful for showing how efficacy signals, safety concerns, and clinical decision-making intersect in practice.

### 5.1 Clinical applications and evidence hierarchy

Classical Chinese medicine has long acknowledged the potent yet toxic nature of *Aconitum carmichaelii* and has therefore relied on formula-based strategies to balance desired effects with safety. Notably, Fuzi is rarely used alone and is more commonly prescribed as one component within multi-herb formulations (Zhou et al., 2015). This clinical context matters when interpreting the literature: reported benefits in circulation, energy support, inflammation, pain, or cardiotonic use often reflect the combined action of the full prescription rather than the isolated effect of Fuzi alone (Deng et al., 2021; Tai et al., 2015; Zhang et al., 2024; Zhao et al., 2012). Thus, classical and modern formula use may support clinical relevance, but it does not justify directly transferring every preclinical claim about single compounds into therapeutic conclusions.

From a clinical-evidence standpoint, however, the literature remains highly heterogeneous and considerably weaker than the mechanistic literature. Only a small subset of reports are prospective randomised studies, Fuzi is usually embedded within a multi-herb intervention, and methodological safeguards such as blinding, allocation concealment, and independent replication are not consistently reported in the studies cited here. Consequently, most of the following formulations should be interpreted as providing supportive or hypothesis-generating evidence rather than definitive proof of efficacy (Cheang et al., 2024; Du et al., 2024; Lina et al., 2025; Song et al., 2012).

#### 5.1.1 Sini decoction

Sini Decoction is composed of one piece of raw Fuzi (15–30 g), Zingiber officinale (Ganjiang), and roasted Glycyrrhiza uralensis (Gancao, licorice) (Tai et al., 2015). It has been reported in clinical contexts such as cardiomyopathy and inflammatory bowel disease (Wang Y. et al., 2021). In a randomised trial of 75 patients with sepsis-associated gastrointestinal dysfunction, acupoint application of Sini Decoction improved enteral nutrition tolerance, motilin levels, and bowel sounds, while reducing abdominal circumference, gastric residual volume, IL-6, lactate, and ICU stay compared with controls. These findings suggest possible benefit in gastrointestinal support during sepsis, although they do not establish Fuzi-specific efficacy independently of the overall intervention (Du et al., 2024). Methodologically, this trial should be viewed as preliminary because the sample size was modest (n = 75), the intervention combined acupoint application with formula therapy, and the reported outcomes were mainly short-term physiological or supportive-care measures. These features limit attribution of effect and reduce confidence that the findings would generalise to broader sepsis populations or translate into major clinical endpoints (Du et al., 2024).

#### 5.1.2 Fuzi Lizhong decoction

Fuzi Lizhong Decoction is composed of one piece of processed Fuzi (15–20 g), Panax ginseng, Zingiber officinale (Ganjiang), Atractylodes macrocephala (Bai Zhu), and roasted Glycyrrhiza uralensis (Gancao, licorice) (Tai et al., 2015). In a randomised study of 100 patients with advanced colorectal cancer, adjunctive use of this decoction with capecitabine plus oxaliplatin was associated with improved gut microbiota balance, lower inflammatory markers, fewer adverse reactions, and better clinical efficacy than chemotherapy alone. These results suggest supportive benefit at the formula level, but they cannot be used to infer efficacy of Fuzi as a single component (Lina et al., 2025). This study also remains exploratory: although randomised, the sample size was still modest (n = 100) for an adjunctive oncology intervention, and the reported benefits were largely biomarker-, microbiota-, and response-based outcomes within a combination regimen. Because Fuzi was not isolated and the robustness of bias-control measures is unclear from the available report, the findings should be interpreted as preliminary supportive evidence rather than conclusive proof (Lina et al., 2025).

#### 5.1.3 Ma Huang Fuzi Xixin decoction

Ma Huang Fuzi Xixin Decoction contains one piece of processed Fuzi (15–20 g), Ephedra sinica (Ma Huang), and *Asarum sieboldii* (Xixin). It has been reported to alleviate allergic rhinitis symptoms by reducing nasal mucosal swelling and lowering IgE and histamine levels (Ding et al., 2024; Wei et al., 2023). It has also been investigated for neuropathic pain relief, although the available evidence should still be interpreted at the level of the overall formula rather than as isolated proof of Fuzi efficacy (Chai et al., 2024). Importantly, the cited allergic-rhinitis and neuropathic-pain studies are predominantly mechanistic or preclinical rather than controlled patient trials. The current evidence therefore supports biological plausibility of the formula, but not robust clinical efficacy in humans (Chai et al., 2024; Ding et al., 2024; Wei et al., 2023).

#### 5.1.4 Zhen Wu decoction

Zhen Wu Decoction consists of Poria cocos (Fu Ling), Paeonia lactiflora Pallas (Shaoyao), fresh Zingiber officinale (ginger), Atractylodes macrocephala (Bai Zhu), and one piece of processed Fuzi (15–20 g) (Tai et al., 2015). It has been used for several kidney-related disorders, including chronic glomerulonephritis. Reported benefits include improved renal function and reduced renal interstitial fibrosis, although these data should still be interpreted within the broader context of formula-based rather than Fuzi-specific effects (Liu et al., 2021; Zheng et al., 2023). However, the references cited here are mainly experimental studies focused on renal mechanisms rather than direct clinical trials in patients. As a result, this formula has plausible translational relevance, but its renal benefit is not yet established by high-quality human evidence (Liu et al., 2021; Zheng et al., 2023).

#### 5.1.5 Guizhi Fuzi decoction

Guizhi Fuzi Decoction includes Cinnamomum cassia (Guizhi), Paeonia lactiflora Pallas (Shaoyao), roasted Glycyrrhiza uralensis (Gancao), fresh Zingiber officinale (ginger), Ziziphus jujuba (jujube), and one piece of processed Fuzi (15–20 g) (Tai et al., 2015). It has traditionally been used for osteoarthritis and other painful musculoskeletal conditions such as rheumatoid arthritis, gouty arthritis, frozen shoulder, and radicular cervical spondylosis (Chen et al., 2023; Jiang et al., 2024). The evidentiary base for this indication appears to rely mainly on traditional use, review-level discussion, and mechanistic or network-pharmacology studies rather than rigorous comparative clinical trials. Clinical claims for osteoarthritis or related pain disorders should therefore be considered provisional (Chen et al., 2023; Jiang et al., 2024).

#### 5.1.6 Fuzi decoction

Fuzi Decoction includes two pieces of processed Fuzi (30–40 g), Poria cocos (Fu Ling), Panax ginseng, Atractylodes macrocephala (Bai Zhu), and Paeonia lactiflora Pallas (Shaoyao) (Tai et al., 2015). It has been discussed for osteoarticular disorders in the traditional literature, and recent mechanistic studies suggest possible chondroprotective, anti-inflammatory, and analgesic actions. However, current support for rheumatoid arthritis and osteoarthritis remains largely preclinical or inference-based rather than firmly established in controlled human trials (Li et al., 2023). This places the current support low on the hierarchy of clinical evidence, and no firm conclusion can yet be drawn about patient-level efficacy, optimal dosing, or long-term safety from the presently cited data (Li et al., 2023).

#### 5.1.7 Qili-qiangxin capsules

Qili-qiangxin capsule is a proprietary herbal preparation developed within a TCM framework for heart failure and approved by the Chinese Food and Drug Administration in 2004 (Tao et al., 2013). Each 0.3 g capsule contains 11 herbs, among which astragalus root and processed Fuzi are major components (Xing et al., 2023). In a large multicentre double-blind RCT (n = 3,110), Qili-qiangxin reduced the risk of heart failure hospitalisation and cardiovascular death in patients with heart failure and reduced ejection fraction, with safety comparable to placebo (Cheang et al., 2024). This is one of the strongest sources of clinical relevance in the literature reviewed here. Even so, the observed benefit relates to the full multi-herb product and should not be attributed solely to Fuzi or its isolated alkaloids. Compared with most other Fuzi-containing interventions reviewed here, this study is methodologically stronger because it was large, multicentre, double-blind, and endpoint-driven. Even so, its findings apply to a standardised proprietary 11-herb product in a specific heart-failure population, so extrapolation to other Fuzi-containing formulas remains limited (Cheang et al., 2024).

#### 5.1.8 Shenfu injection

Shenfu Injection is a Chinese medicinal preparation made from red ginseng and processed Fuzi and has been widely discussed for use in severe conditions such as heart failure and septic shock (Yang et al., 2014). The injection contains 676–742 μg/mL of ginsenosides and 3–7 μg/mL of aconite alkaloids (Xu et al., 2024). A systematic review of 99 RCTs reported improvements in heart function, NT-proBNP levels, exercise capacity, and clinical response rate when Shenfu Injection was added to routine therapy in heart failure patients (Song et al., 2012). However, most trials were of limited methodological quality. Therefore, Shenfu Injection provides suggestive but not definitive clinical support, and its results should again be interpreted at the level of the formulation rather than as direct proof of Fuzi-alone efficacy. Accordingly, the apparent consistency across many studies should not be overinterpreted, because meta-analytic precision cannot offset systematic bias in the underlying trials. The large number of included RCTs therefore increases interest in the formulation, but not certainty to the level provided by a well-conducted contemporary multicentre trial (Song et al., 2012).

Taken together, the clinical evidence is clearly tiered rather than uniform. Qili-qiangxin provides the most robust human data in this review; Shenfu Injection offers suggestive but downgraded evidence because the pooled trials were methodologically weak; Sini Decoction and Fuzi Lizhong Decoction are supported mainly by smaller adjunctive studies; and several other formulas remain backed largely by preclinical or narrative literature. Making this hierarchy explicit is important to avoid overstating clinical certainty (Chai et al., 2024; Cheang et al., 2024; Du et al., 2024; Lina et al., 2025; Song et al., 2012). The clinical evidence hierarchy for selected Fuzi-containing preparations is summarised in Table 1.

**TABLE 1:** Clinical evidence hierarchy for selected Fuzi-containing preparations discussed in this review.

Preparation/intervention	Highest evidence cited	Clinical context	Main findings reported	Interpretation/limitations
Qili-qiangxin capsules	Large multicentre double-blind RCT (n = 3,110)	Heart failure with reduced ejection fraction	Reduced heart-failure hospitalisation and cardiovascular death with safety comparable to placebo (Cheang et al., 2024)	Strongest human evidence in this review, but applies to a standardised 11-herb product rather than Fuzi alone
Shenfu Injection	Systematic review of 99 RCTs	Heart failure and severe cardiovascular conditions	Improved heart function, NT-proBNP, exercise capacity and response rate (Song et al., 2012)	Suggestive but downgraded because many underlying trials had methodological limitations; formulation-level evidence
Sini Decoction	Small randomised clinical study (n = 75)	Sepsis-associated gastrointestinal dysfunction	Improved enteral nutrition tolerance and short-term physiological outcomes (Du et al., 2024)	Preliminary adjunctive evidence; acupoint/formula intervention limits attribution to Fuzi
Fuzi Lizhong Decoction	Small randomised adjunctive study (n = 100)	Advanced colorectal cancer with chemotherapy	Reported effects on microbiota, inflammatory markers, adverse reactions and clinical response (Lina et al., 2025)	Exploratory formula-level evidence; blinding and Fuzi-specific contribution remain uncertain
Ma Huang Fuzi Xixin Decoction	Mainly preclinical/mechanistic literature	Allergic rhinitis and neuropathic pain models	Biological plausibility through inflammatory, epithelial barrier, and pain-signalling pathways (Chai et al., 2024; Ding et al., 2024)	Not robust clinical evidence; human efficacy remains insufficiently established
Zhen Wu, Guizhi Fuzi and Fuzi Decoctions	Mostly experimental, network-pharmacology, or narrative evidence	Renal and osteoarticular indications	Mechanistic signals for renal fibrosis, mitochondrial function, inflammation and pain (Chen et al., 2023; Liu et al., 2021; Zheng et al., 2023)	Low certainty for patient-level efficacy; controlled clinical trials are needed

### 5.2 Poisoning incidents

Aconitine, being the main toxic substituent in *Aconitum carmichaelii*, can cause human poisoning at a dose of 0.2 mg, with 2–4 mg being fatal (Jiang et al., 2023). Therefore, intoxication cases with *Aconitum carmichaelii* remain a global concern. Between 2001 and 2010, around 5,000 aconite poisoning cases were reported worldwide, spanning countries like China, Japan, Germany, and others (Yang M. et al., 2018). More than 41 aconite poisoning cases were recorded between 2012 and 2017 in Hong Kong, whereas in Mainland China, at least 40 cases of fatal aconite poisoning involving 53 victims were documented between 2003 and 2015 (Li et al., 2016). In Hong Kong, while the data on the exact doses used for each poisoning case is not retrievable, a review reported overdosing (recommended doses: Chuanwu = 1.5–3g, Fuzi = 3–15 g) and inappropriate processing of aconite roots as the main causes of poisoning (Chan, 2015). In China, a retrospective analysis found that consumption of medicinal liquors containing aconitine was the most frequently reported (62%) exposure route, followed by cooking (23%) and herbal medicine (15%), as ethanol can prompt aconitine absorption into the blood. This combination can produce synergistic toxicity due to central nervous system inhibition and neurological symptoms (Li et al., 2016). Other causes of toxicity include improper self-prescription, mistaken ingestion, contamination of other herbs, and even intentional use in suicides and homicides (Yang M. et al., 2018). Poisoning cases were reported in Southeast Asia as well. A 34-year-old postpartum woman in Malaysia who had been consuming an aconitum-containing herbal pack during confinement developed diarrhoea, perioral and limb numbness, hypotension, and bradycardia. She improved after IV atropine, activated charcoal, and fluid resuscitation, requiring 1 day of ICU care and discharge after 3 days (Lau and Azman, 2021). In Singapore, a 34-year-old man who ingested a recipe containing 30 g of Chuanwu for pain relief developed numbness throughout his body, weakness, and shortness of breath. He was subsequently rushed to the hospital and recovered following treatment (Sheth et al., 2015).

Notably, the wild herb is more toxic than the processed drug, thus, the poisoning cases in the past were usually severe and fatal (Li et al., 2016). Patients with aconite poisoning typically manifest with nausea, vomiting, dizziness, hypotension, palpitations, arrhythmias, shock, and even coma, reporting an average onset time of about 44 min (Li et al., 2016; Zhou et al., 2015). Ventricular tachycardia, fibrillation, premature contractions, multifocal ectopics, sinus tachycardia, and bradycardia are among the ECG abnormalities. Fatal outcomes usually result from ventricular arrhythmias and cardiac arrest, most commonly within the first 24 h after ingestion (Yang M. et al., 2018). As no specific antidote exists, management relies on intensive cardiovascular support, in which inotropic therapy is indicated if hypotension remains and bradycardia should be treated with atropine (Chan, 2009). Nevertheless, clinical manifestations vary with dose and patient condition even though no clear dose-response relationship has been documented (Li et al., 2016; Singhuber et al., 2009).

### 5.3 Clinical-toxicological interpretation of poisoning risk

The poisoning literature provides the strongest translational link between mechanistic toxicology and real-world clinical outcomes. Reported cases indicate that risk is shaped by three interacting exposure determinants: total alkaloid load, preparation route, and patient vulnerability. Alcoholic preparations and medicinal liquors are especially concerning because ethanol can extract aconite alkaloids and may increase systemic exposure, whereas decoctions are influenced by processing quality, boiling duration, and whether raw or processed material was used. Proprietary products add a separate risk of adulteration, substitution, or inconsistent batch composition. Exact dose-response relationships are difficult to reconstruct from case reports because many do not quantify the ingested alkaloid content, but the rapid onset of paresthesia, gastrointestinal symptoms, hypotension, bradycardia or tachyarrhythmia, and ventricular arrhythmias is consistent with the sodium-channel and calcium-overload mechanisms described in toxicodynamic studies (Chan, 2009; Li et al., 2016; Yang M. et al., 2018).

## 6 Safety considerations

While *Aconitum carmichaelii* has shown remarkable therapeutic potential, its clinical application is constrained by a very narrow therapeutic margin and risks of cardiotoxicity and neurotoxicity. Ensuring safe use requires not only strict dosage control but also appropriate detoxification techniques, rational herb-herb combinations, and careful patient monitoring. Safety strategies therefore aim not to abolish pharmacological activity altogether, but to keep these overlapping efficacy-toxicity mechanisms within a manageable therapeutic range.

### 6.1 Detoxification protocols

The therapeutic use of Fuzi in TCM requires detoxification prior to application because the raw herb contains higher levels of toxic DDAs and appropriate processing substantially reduces their concentration. Despite such measures, the diversity of processing methods and the herb’s narrow therapeutic index mean that poisoning may still occur (Nyirimigabo et al., 2015). Traditional detoxification techniques primarily hydrolyse toxic DDAs into less toxic MDAs or ADAs through removal of ester groups at the C8 and C14 positions, resulting in safer derivatives while retaining part of the desired pharmacological activity (Yang M. et al., 2018). For example, processing converts aconitine into less toxic derivatives such as benzoylaconine and aconine; however, these products should be interpreted as lower-risk rather than risk-free. Processed forms such as Baifupian, Heishunpian, and Yanfuzi therefore exhibit markedly reduced toxicity compared with crude Fuzi, but their safety still depends on validated processing, dosage control, and analytical confirmation of residual toxic alkaloids (Chan et al., 2021; Gao et al., 2022; Tong et al., 2013).

#### 6.1.1 Boiling

Boiling is the simplest and most widely used method of detoxification. Clinical and analytical data demonstrate that longer boiling times (10–120 min) progressively hydrolyse DDAs into MDAs and ADAs. For instance, Baifupian boiled for two hours exhibited no measurable toxicity in mice, with aconitine undetectable by HPLC, whereas shorter boiling times (30–60 min) retained residual toxicity (Chan et al., 2021). Interestingly, total alkaloid content remains relatively stable, indicating transformation rather than elimination of compounds (Chan et al., 2021). Importantly, therapeutic efficacy is preserved across boiling durations. However, boiling may result in the loss of some beneficial constituents due to water solubility (Liu et al., 2017).

#### 6.1.2 Baking/cooking

Baking or stir-frying represents another traditional approach, often yielding Fuzi preparations with safe levels of DDAs but variable MDA content. Paofuzi, for example, shows very low DDA levels but also reduced MDAs, potentially compromising efficacy (Chan et al., 2021; Wen et al., 2013). Comparative studies of dry-baking, modern baking, stir-frying, and sand-burning methods demonstrated that processed Zhifuzi contained 3–4 times higher MDA content than crude Fuzi while maintaining low DDA levels. Another study showed that steaming or stir-frying can reduce DDA content by 40–80 times, regardless of saline use. However, saline processing may limit the formation of MDAs (Wen et al., 2013). Given their retained activity and lower toxicity, the MDA/DDA ratio has been proposed as a quality control marker for processed Fuzi (Chan et al., 2021).

#### 6.1.3 Steaming

Steaming or autoclaving has emerged as a fast, clean, cost-effective, and easily standardized detoxification technique. Crude Fuzi is steamed at 127 °C under 0.15 MPa pressure for 60–90 min as an optimal window balancing efficacy and safety (Liu et al., 2006). This method efficiently converts toxic compounds into safer derivatives while preserving the total alkaloid content and pharmacological activity (Chan et al., 2021).

### 6.2 Herb-herb combinations

Nevertheless, two major concerns remain. First, poisoning may still occur even after processing Fuzi, as toxic alkaloids can persist at hazardous levels or be ingested in excessive amounts (Huang et al., 2007). Second, the therapeutic effects of Fuzi are closely linked to the presence of these toxic alkaloids, meaning removal of them can potentially diminish the herb’s overall therapeutic efficacy (Tong et al., 2013). Nowadays, individual patterns of syndrome differentiation and specifically tailored treatment plans often demand multi-herb formulas which not only target various symptoms but also balance the formula’s energetic properties. Compatibility between herbs can further enhance efficacy and reduce toxicity.

#### 6.2.1 Ginseng (*Panax notoginseng*)

Ginseng acts synergistically with Fuzi as both enhance efficacy and reduce toxicity. This combination can increase superoxide dismutase activity and decrease the content of malondialdehyde, nitrogen oxide, and lactate dehydrogenase, thus improving cardiac cell viability (Yang L. et al., 2018). Ginseng also regulates CYP2J3, CYP4A3, and CYP4F11, protecting cardiac cells and reducing LD50 more effectively than Licorice (Chan et al., 2021).

#### 6.2.2 Gancao (*radix Glycyrrhizae*, licorice)

Gancao has long been used in combination with Fuzi for its detoxifying ability as it stimulates the function of CYP3A4 and CYP2B6, facilitating aconitine efflux via P-glycoprotein (He et al., 2019). Compounds such as glycyrrhizin and glycyrrhetinic acid provide strong antioxidant protection that reduces lipid peroxidation and oxidative stress on cardiac cells. Additionally, glycyrrhetinic acid increases anti-apoptotic Bcl-2 expression and suppresses pro-inflammatory cytokines, thereby protecting cardiac tissue (Chan et al., 2021). This combination can also help reduce inflammation and ventricular remodelling via the TLR4/NF-κB signalling pathway (Yan et al., 2020).

#### 6.2.3 Ganjiang (*Zingiberis Rhizoma*, dried ginger)

Ganjiang can enhance therapeutic outcomes of Fuzi by promoting mitochondrial biogenesis via Sirt1, PGC-1α, and NRF1 pathways, which aid in the treatment of heart failure (Lu et al., 2017). It also reduces oxidative stress markers like hydrogen peroxide and malondialdehyde and increases superoxide dismutase activity. The combination improves hemodynamics and suppresses neuroendocrine dysregulation more effectively than Fuzi alone (Chan et al., 2021).

#### 6.2.4 Dahuang (Rhei radix et Rhizoma)

When used in combination with Fuzi, Dahuang markedly diminishes the levels of toxic diester DDAs, alleviating arrhythmia-related side effects such as ventricular tachycardia in a dose-dependent way. When the concentration of Dahuang is higher relative to Fuzi, the alkaloid toxicity is lower (Chan et al., 2021).

### 6.3 Clinical precautions

In an attempt to ensure safe and effective use of Fuzi in clinical settings, several key precautions including rational dosage guidelines and patient-specific contraindications must be considered.

#### 6.3.1 Dosage guidelines

Based on the ChP, the safe clinical dosage of Fuzi is 3–15 g/person/day, provided that it is decocted first (Chinese Pharmacopoeia Commission, 2025). However, this recommendation lacks bench evidence and does not differentiate between the different processing types of Fuzi like Heishunpian and Paofupian (Ji et al., 2023). Notably, the active constituent, aconitine, exhibited cardiotonic effects solely at lower concentrations and its effectiveness could be negated by the arrhythmia caused by the elevated aconitine content (Liu et al., 2012). Therefore, it has been proposed that the effects of aconitine can differ based on the dosage, akin to cardiac glycosides, warranting accurate dosage adherence (Zhao et al., 2024).

#### 6.3.2 Contraindications

Both the ChP and the Taiwan Herbal Pharmacopoeia (THP) advise caution against the use of processed Fuzi during pregnancy (Chinese Pharmacopoeia Commission, 2025; Ministry Health and Welfare, 2022). Moreover, patients with severe liver and renal impairments, pre-existing arrhythmias, or advanced cardiovascular diseases may be at increased risk of adverse effects due to impaired clearance and heightened cardiac sensitivity. Therefore, close monitoring and professional supervision are crucial.

### 6.4 Risk-benefit framework for clinical decision-making

A practical risk-benefit assessment should consider not only whether Fuzi has plausible pharmacological effects, but also whether the intended preparation, dose, patient population, and monitoring strategy keep exposure within a defensible safety margin. In this framework, stronger benefit claims require stronger human evidence and better standardisation, whereas safety controls should be applied even when efficacy evidence is preliminary because severe toxicity can occur rapidly and unpredictably. These considerations are organised into a practical risk-benefit framework in Table 2.

**TABLE 2:** Practical risk-benefit framework for interpreting Fuzi use.

Assessment domain	Potential benefit signal	Main risk signal	Practical control
Alkaloid exposure	Cardiotonic or analgesic signals in controlled preclinical settings	Arrhythmia, neurotoxicity and cardiovascular collapse when DDAs predominate	Use processed material; quantify DDA/MDA markers; avoid excessive dose or alcohol extraction
Preparation and processing	Hydrolysis may retain some pharmacological activity while reducing toxicity	Incomplete processing leaves toxic DDAs; excessive processing may reduce active constituents	Document processing method; use validated HPLC/UHPLC assays and batch specifications
Patient factors	Possible supportive role in selected supervised indications	Pregnancy, arrhythmia, severe cardiovascular disease, hepatic or renal impairment increase vulnerability	Screen contraindications; monitor ECG and symptoms when clinically justified
Evidence threshold	Large controlled trials support selected proprietary formulations	Most decoction or isolated-compound claims remain low certainty	Align clinical claims with evidence level; avoid extrapolating preclinical mechanisms to patient benefit

## 7 Regulatory challenges

The strong toxicity and limited therapeutic margin of *Aconitum carmichaelii* have made its current regulation a top priority for pharmacopoeial committees and public health authorities in recent decades. A study using HPLC reported a significant fluctuations in alkaloidal contents among four different species of Aconitum, namely, *Aconitum carmichaelii*, *Aconitum pendulum*, *Aconitum hemsleyanum*, and *Aconitum transsectum*, underscoring the significance of selecting the correct species with known alkaloid levels for herbal remedies (Wang Z. et al., 2006).

### 7.1 Quality control and analytical standards

The quality control must include both qualitative and quantitative analyses to confirm proper processing and detoxification of the raw herbs. Among the available methods, liquid chromatography is the “gold standard” in TCM quality control and plays a vital role in assessing and regulating Aconitum-based formulations. According to the ChP, the moisture content in processed Fuzi should be below 15%. Using isopropanol-ethyl acetate as the solvent, HPLC is employed to quantify the levels of alkaloids. Additionally, the content of DDAs must not exceed 0.020% while the content of MDAs must be at least 0.010% (Chinese Pharmacopoeia Commission, 2025).

### 7.2 Country-specific regulations

Nevertheless, regulatory standards for Aconitum-containing products vary globally, necessitating standardisation. Selected regulatory approaches across jurisdictions are compared in Table 3.

**TABLE 3:** Comparison of selected regulatory approaches for Aconitum-containing materials and products.

Jurisdiction	Regulatory object	Key alkaloid or processing control	Implication for safety governance
China	Processed Fuzi recognised in ChP	DDA <= 0.020%; MDA >= 0.010%; processed forms and 3–15 g/day dosage guidance	Specific pharmacopoeial limits support standardisation but clinical exposure-response data remain limited
Taiwan	Processed Fuzi in Taiwan Herbal Pharmacopoeia	MDA > 0.01%; DDA < 0.02%; processed oral use 3–15 g	Broadly aligned with ChP; pregnancy caution remains explicit
Japan	Processed aconite root in Japanese Pharmacopoeia	Total alkaloids expressed as benzoylaconine vary by processing method	Regulation differentiates processing processes and permitted alkaloid ranges
Korea	Prepared daughter root in Korean Pharmacopoeia	Total alkaloid content estimated as benzoylaconine should not exceed specified limit	Uses prepared-product control but differs from ChP marker strategy
India	Aconitum herbs under Schedule E (1) poison framework	Traditional detoxification (Shodhana) before medicinal use	Professional supervision and processing are central safety requirements
Australia	Poisons Standard and ARTG product oversight	Adult pack limits and prescription classification depending on alkaloid content	Finished-product testing can trigger recalls when alkaloid limits are exceeded
Malaysia/Singapore	Traditional product restrictions or daily alkaloid limits	Malaysia treats aconite alkaloids as scheduled poisons; Singapore limits daily alkaloid exposure	Highlights the need for finished-product surveillance in Southeast Asia
US/Europe/Canada	Variable frameworks for raw herbs, products and homeopathic preparations	No unified Aconitum-specific pharmacopoeial limit	Risk depends on enforcement, product category, import control and post-market detection

#### 7.2.1 China

The ChP allows only the usage of processed roots with strict alkaloid thresholds mentioned in Section 7.1, while prohibiting the use of raw herb due to its high toxicity. The recognised processed forms include Yanfuzi (salt-processed), Heishunpian (black sliced), Baifupian (white sliced). The recommended clinical dosage of processed Fuzi is 3–15 g/day/person along with the standardised processing procedures (soaking, steaming, prolonged boiling) to ensure detoxification.

#### 7.2.2 Taiwan

Taiwan adopts a similar standard to China, the processed Fuzi can be categorised into Yanfuzi, Heishunpian, and Baifupian with soaking, steaming, and prolonged boiling as the processing methods. Aligned with the ChP, the key safety limitations stated in THP include thresholds of MDA level larger than 0.01% and DDA less than 0.02% to guarantee safe clinical usage. The processed form is orally administered at a dosage of 3–15 g (Ministry Health and Welfare, 2022).

#### 7.2.3 Japan

The processed aconite root obtained from the tuberous root of *Aconitum carmichaelii* is listed in the Japanese Pharmacopoeia. It is prepared using three major processing techniques, which are autoclaving (Process 1), salt solution rinsing followed by heating or autoclaving (Process 2), and salt solution rinsing followed by calcium hydroxide treatment (Process 3). Each method produces a different permitted range of total alkaloid concentration, expressed as benzoylaconine: 0.7%–1.5% for Process 1, 0.1%–0.6% for Process 2, and 0.5%–0.9% for Process 3, calculated on the dried base (Pharmaceutical and Medical Device Regulatory Science Society of Japan, 2021). These processing methods are critical to cleanse the aconite root while standardising its alkaloid content to ensure safe therapeutic use.

#### 7.2.4 Korea

The Korean Pharmacopoeia classified the prepared daughter root of *Aconitum carmichaelii* into three varieties based on the preparation methods, namely, Yeombuja (salted aconite), Bujapyeon (sliced aconite), and Pobuja (boiled aconite) (Ministry of Food and Drug Safety, 2020). The Korean authority requires that the total alkaloid content estimated as benzoylaconine in prepared aconite not exceed 0.33% using the titration method (Ministry of Food and Drug Safety, 2015).

#### 7.2.5 India

Although *Aconitum carmichaelii* is not as significantly mentioned as local Indian species like *Aconitum ferox* (Vatsanabha), Aconitum herbs are Schedule E (1) poisons under the Drugs and Cosmetics Rules, 1945, meaning it is strictly forbidden to take any medication containing them without the supervision of licensed Ayurvedic practitioners (Ministry of Health and Family Welfare, 2016). The Ayurvedic Pharmacopoeia of India emphasises the need to process (Shodhana) any Aconitum root using traditional detoxifying techniques, such as boiling it in cow’s milk or creating herbal decoctions, before utilising it medicinally (Jaiswal et al., 2013).

#### 7.2.6 Australia

*Aconitum carmichaelii* and related species are governed in Australia by the Therapeutic Goods Administration (TGA) under the Poisons Standard (SUSMP). Depending on their intended use and alkaloid concentration, they are listed in both Schedules 2 and 4. While Schedule 2 permits limited usage in adults only when the total alkaloids per pack fall between 0.02 mg and 0.2 mg, Schedule 4 applies generally and classifies Aconitum as a Prescription Only Medicine, which also allows children use. Any product that exceeds these limitations or does not have the appropriate adult-only labelling falls under Schedule 4 (Australian Government, 2025). TGA testing of 19 medications containing Aconitum registered on the Australian Register of Therapeutic Goods revealed that the sample of Jin Gui Shen Qi Wan had an alkaloid content (aconitine, hypaconitine and mesaconitine) of 0.3 mg/pack, which was higher than allowed, leading to product recall (Australian Government, 2021).

#### 7.2.7 Western countries

In the United States, Canada, and much of Europe, *Aconitum carmichaelii* should not be described simply as universally prohibited. Rather, it is generally absent from official pharmacopoeial monographs and is treated cautiously or restrictively because of toxicity, while different product categories-such as raw herbal material, finished herbal products, imported TCM formulas, and highly diluted homeopathic preparations-may fall under different legal frameworks. This distinction is important because the regulatory challenge is not only whether aconite is permitted, but also whether species identity, processing status, residual alkaloid content, finished-product quality, and post-market surveillance can be verified consistently (Singhuber et al., 2009; Tai et al., 2015).

#### 7.2.8 Southeast Asian countries

While biomedicine and TCM are equally recognized in Malaysia, the country classifies such medicines based on the specific toxic substance. Aconite and its alkaloids are categorised as a scheduled poison under the Poisons Act 1952, prohibiting their usage in traditional medicines. In 2015, the traditional product Li Chung Pill was found to be adulterated with the scheduled poisons aconitine, mesaconitine, and hypaconitine, resulting in the cancellation of its product registration (National Pharmaceutical Regulatory Agency, 2016). On the other hand, the government of Singapore imposes a dosing limit of no more than 60 μg per day for aconite and its alkaloids in Chinese Proprietary Medicines (Health Sciences Authority Government of Singapore, 2025).

### 7.3 Enforcement challenges in standardization

*Aconitum carmichaelii* has a narrow therapeutic margin, and its safe clinical use depends on standardised preparation, validated quality control, appropriate clinical indication, and enforceable regulatory oversight. Although the ChP 2025 and other regional standards provide important frameworks, several challenges continue to limit consistent preparation, comparable quality control, and global acceptance of Fuzi-containing products.

#### 7.3.1 Processing variability

DDAs (aconitine, mesaconitine, and hypaconitine) are the main contributors to acute aconite toxicity and should be transformed into less toxic products through validated processing (Singhuber et al., 2009). However, different manufacturers and geographical regions adopt different processing techniques, resulting in variable alkaloid profiles. HPLC-TOF/MS studies have reported aconitine levels in processed Fuzi ranging from undetectable to 0.02%, indicating inconsistent detoxification (Liu et al., 2017). From 2004 to 2009, 20 poisoning cases in Hong Kong were linked to inappropriate processing that caused severe neurological and cardiovascular symptoms (Chen et al., 2012). At the same time, excessive degradation of pharmacologically relevant alkaloids such as benzoylaconine may diminish potential efficacy (Zhang et al., 2022). This dual concern explains why quality control should assess both toxicity reduction and retention of defined marker compounds rather than focusing only on absence of DDAs.

#### 7.3.2 Analytical limitations

Accurate quantification of alkaloid content is central to quality control of *Aconitum carmichaelii*. The ChP recommends measuring six key alkaloids-aconitine, mesaconitine, hypaconitine, benzoylaconine, benzoylmesaconine, and benzoylhypaconine-using HPLC (Chinese Pharmacopoeia Commission, 2025). More advanced UHPLC-MS/MS methods offer greater sensitivity, but these platforms may not be accessible to smaller laboratories or routine market-surveillance settings (Zhang et al., 2020). Additional analytical challenges include discrepancies between methods such as capillary electrophoresis and HPLC, matrix interference from polysaccharides and phenolic compounds, and the need for stability-indicating assays because alkaloids can transform during processing and storage (Csupor et al., 2011; Song et al., 2010). Simpler methods such as thin-layer chromatography may be useful for screening but lack the precision required for regulatory compliance.

#### 7.3.3 Hidden alkaloids, stability and batch-to-batch control

Quality control should also address constituents that may be underestimated by routine marker-based assays. Lipo-alkaloids and other masked or matrix-bound alkaloid forms can complicate interpretation because they may transform during processing, storage, decoction, or digestion. Batch-to-batch control should therefore include species authentication, origin and harvest documentation, processing records, moisture control, DDA/MDA ratio assessment, residual toxic alkaloid limits, and finished-product testing. These measures are particularly important for proprietary products and exported preparations, where the material assessed by regulators may differ from the raw herb originally described in traditional texts.

#### 7.3.4 Clinical data gaps

Modern clinical studies on *Aconitum carmichaelii* remain limited despite the herb’s long history of use. The ChP recommendation of 3–15 g/day Fuzi is not strongly supported by RCT-based dose-response evidence (Ji et al., 2023). Most pharmacokinetic work has been conducted in animals, whereas human pharmacokinetic and exposure-response data remain sparse (Zhang et al., 2020). This imbalance affects interpretation of the pharmacological literature because many proposed therapeutic actions are based on isolated compounds, cell systems, or rodent models that differ substantially from processed clinical use and multi-herb prescribing contexts. Given its cardiotoxic potential, long-term safety, cumulative exposure, herb-drug interactions, and population-specific vulnerability remain unresolved clinical questions (Chan, 2009).

#### 7.3.5 Global harmonisation

Global harmonisation is difficult because jurisdictions regulate different objects and use different analytical endpoints. Some standards focus on processed raw materials, whereas others regulate finished products, total alkaloids, selected DDA/MDA markers, daily exposure, or prescription-only status. Expression units also vary, for example, individual marker percentages versus benzoylaconine-equivalent total alkaloids. A feasible harmonisation strategy would therefore be to agree on minimum safety principles-species authentication, validated processing, DDA/MDA marker quantification, finished-product surveillance, contraindication labelling, and adverse-event reporting-rather than imposing a single universal alkaloid limit across all preparations (Chan, 2009; Singhuber et al., 2009).

### 7.4 Distinctive contribution relative to recent reviews

Because lack of novelty is a legitimate concern for a topic with many existing reviews, the contribution of the present manuscript is explicitly defined against the recent review landscape. Li et al. (2025) uses integrative toxicology to explain how TCM toxicity-efficacy concepts can be reconciled with modern toxicology (Li et al., 2025). The present article instead contributes a clinical-regulatory synthesis that connects mechanistic plausibility to human poisoning, evidence hierarchy, analytical quality control, product surveillance and international governance. These complementary but non-overlapping emphases are summarised in Table 4.

**TABLE 4:** Distinctive contribution of the present clinical-regulatory review compared with the recent integrative-toxicology review by Li et al. (2025).

Domain	Li et al. (2025)	Present review
Conceptual foundation	Integrative toxicology; bridges TCM toxicity-efficacy theory with modern toxicology	Clinical-regulatory risk-benefit synthesis for safer interpretation, product control and governance
Evidence integration	Emphasises chemistry, pharmacological activity, toxicity mechanisms, processing and compatibility	Adds explicit evidence hierarchy, human poisoning patterns, proprietary formulations, clinical-toxicological interpretation and regulatory implications
Clinical relevance	Primarily mechanistic and theoretical, with limited emphasis on real-world poisoning management or clinical evidence ranking	Links ion-channel, mitochondrial and inflammatory toxicology to poisoning cases, exposure route, onset pattern, management challenges and patient vulnerability
Regulatory scope	Focuses mainly on the Chinese context and pharmacopoeial detoxification principles	Compares China, Taiwan, Japan, Korea, India, Australia, Southeast Asia, North America and Europe, including alkaloid limits, finished-product surveillance and analytical challenges
Practical output	Provides a conceptual framework for understanding toxicity-efficacy transformation	Provides a practice-oriented framework for clinicians, toxicologists, pharmacologists, manufacturers and regulators

## 8 Limitations and future research

Several limitations remain in the current evidence base on *Aconitum carmichaelii*. First, most pharmacological and toxicological data are derived from *in vitro* experiments or animal models, and many experimental concentrations are not clearly linked to clinically achievable exposure after processed Fuzi use. Second, many pharmacodynamic findings are reported for isolated alkaloids or multi-herb formulas rather than standardised Fuzi preparations, making Fuzi-specific attribution difficult (Bachhar et al., 2026; Bachhar et al., 2025b; Haldhar et al., 2025; Joshi et al., 2025c). Third, dose-response relationships, human pharmacokinetics, and long-term safety remain inadequately characterised. Fourth, processing variability and batch-to-batch differences in alkaloid profiles complicate both efficacy interpretation and toxicological risk assessment (Bachhar et al., 2025a; Joshi et al., 2025a). Fifth, regional regulatory standards differ in marker compounds, units of measurement, permitted product categories, and finished-product surveillance, limiting international comparability (Chan, 2009; Ji et al., 2023; Singhuber et al., 2009; Wu et al., 2018; Zhang et al., 2020).

Future research should prioritise a clearer translational agenda. Well-designed clinical trials should evaluate processed Fuzi or Fuzi-containing formulas under defined indications, with transparent reporting of botanical identity, processing method, alkaloid profile, dose, co-medications, adverse-event monitoring, and clinically meaningful outcomes. Human pharmacokinetic and exposure-response studies are needed to determine whether proposed mechanisms occur at relevant concentrations and to identify vulnerable populations. Translational models should use clinically processed preparations, formula-based comparisons, human-relevant cell systems, and safety endpoints that align with known poisoning mechanisms (Joshi et al., 2025b; Joshi et al., 2026). Finally, pharmacopoeial and regulatory systems should integrate sensitive trace-alkaloid analytics, stability testing, finished-product surveillance, and harmonised adverse-event reporting to support safer global use.

## 9 Conclusion

*Aconitum carmichaelii* remains a classical but high-risk medicinal herb because its potential therapeutic actions and toxic effects are both closely linked to diterpenoid alkaloid chemistry. Experimental evidence supports plausible cardiovascular, anti-inflammatory, analgesic, immunomodulatory, and anti-tumour mechanisms, but the strength of clinical evidence differs substantially across indications and preparations. The most credible human support currently comes from standardised proprietary formulations tested in larger controlled trials, whereas many traditional decoctions and isolated-compound claims remain supported mainly by preclinical, adjunctive, or methodologically limited studies. In contrast, the hazards of cardiotoxicity, neurotoxicity, arrhythmia, and fatal poisoning are well documented when exposure is excessive, processing is inadequate, or product quality is uncertain. The distinctive contribution of this review is its clinical-regulatory framing: it links mechanistic toxicology to real-world poisoning, evidence hierarchy, product quality, analytical control, and international safety governance rather than simply repeating prior phytochemical or integrative-toxicology summaries. Safe interpretation of Fuzi therefore requires a risk-benefit approach that combines evidence hierarchy, validated processing, marker-based alkaloid quantification, finished-product quality control, patient-specific contraindication assessment, and regulatory surveillance. Future progress depends on human pharmacokinetic studies, rigorous clinical trials, stability-aware analytical standards, and greater international harmonisation of safety requirements.

## References

[B1] Ameri A. (1998). The effects of *Aconitum* alkaloids on the central nervous system. Prog. Neurobiol., 56(2), 211–235. 10.1016/S0301-0082(98)00037-9 9760702

[B2] Australian Government (2021). Aconitum Alkaloids in Listed Medicines. Available online at: https://www.tga.gov.au/resources/publication/tga-laboratory-testing-reports/aconitum-alkaloids-listed-medicines (Accessed March 7, 2026).

[B3] Australian Government (2025). Therapeutic Goods (Poisons Standard—June 2025) Instrument 2025. Available online at: https://www.legislation.gov.au/F2025L00599/latest/text (Accessed March 7, 2026).

[B4] Bachhar V. Joshi V. Bhatia A. Rom T. Duseja M. Shukla R. K. (2025a). Green synthesis of AgFe bimetallic nanoparticles from Calyptocarpus vialis plant extract for enhanced catalytic reduction of 4-NP, antioxidant and antibacterial activities. J. Environ. Chem. Eng. 13(3), 116829. 10.1016/j.jece.2025.116829

[B5] Bachhar V. Joshi V. Singh P. Berisha A. Haldhar R. Duseja M. (2025b). Anticorrosion and rheological properties of Calyptocarpus vialis extract as a green coating for mild steel in 2MH2SO4. Colloids Surfaces A Physicochem. Eng. Aspects, 705, 135606. 10.1016/j.colsurfa.2024.135606

[B6] Bachhar V. Joshi V. Haldhar R. Kim S.-C. Duseja M. Katin K. P. (2026). A sustainable triple-action inhibitor: corrosion protection, antioxidant activity, and antibacterial performance of Solanum chrysotrichum fruit extract. Bioresour. Technol., 439, 133385. 10.1016/j.biortech.2025.133385 41016557

[B7] Bi C. Zhang T. Li Y. Zhao H. Zhang P. Wang Y. (2020). A Proteomics- and metabolomics-based study revealed that disorder of palmitic acid metabolism by Aconitine induces cardiac injury. Chem. Res. Toxicol. 33 (12), 3031–3040. 10.1021/acs.chemrestox.0c00372 33236894

[B8] Catterall W. A. (1988). Structure and function of voltage-sensitive ion channels. Science 242 (4875), 50–61. 10.1126/science.2459775 2459775

[B9] Chai Y. He S. Liang D. Gu C. Gong Q. Long L. (2024). Mahuang Fuzi Xixin decoction: a potent analgesic for neuropathic pain targeting the NMDAR2B/CaMKIIα/ERK/CREB pathway. Heliyon 10 (16), e35970. 10.1016/j.heliyon.2024.e35970 39211918 PMC11357756

[B10] Chan T. Y. (2009). Aconite poisoning. Clin. Toxicol. (Phila) 47 (4), 279–285. 10.1080/15563650902904407 19514874

[B11] Chan T. Y. (2015). Incidence and causes of aconitum alkaloid poisoning in Hong Kong from 1989 to 2010. Phytotherapy Res. 29 (8), 1107–1111. 10.1002/ptr.5370 25974837

[B12] Chan Y. T. Wang N. Feng Y. (2021). The toxicology and detoxification of *Aconitum*: traditional and modern views. Chin. Med. 16 (1), 61. 10.1186/s13020-021-00472-9 34315520 PMC8314510

[B13] Cheang L. Yao W. Zhou Y. Zhu X. Ni G. Lu X. (2024). The traditional Chinese medicine Qiliqiangxin in heart failure with reduced ejection fraction: a randomized, double-blind, placebo-controlled trial. Nat. Med. 30 (8), 2295–2302. 10.1038/s41591-024-03169-2 39095596 PMC11333273

[B14] Chelko S. P. Asimaki A. Lowenthal J. Bueno-Beti C. Bedja D. Scalco A. (2019). Therapeutic modulation of the immune response in arrhythmogenic cardiomyopathy. Circulation 140 (18), 1491–1505. 10.1161/circulationaha.119.040676 31533459 PMC6817418

[B15] Chen S. P. Ng S. W. Poon W. T. Lai C. K. Ngan T. M. Tse M. L. (2012). Aconite poisoning over 5 years: a case series in Hong Kong and lessons towards herbal safety. Drug Saf. 35 (7), 575–587. 10.2165/11597470-000000000-00000 22631223

[B16] Chen D. T. Shen X. Li Y. M. Chen L. Pan Y. B. Sheng X. P. (2023). To explore the mechanism of “Fuzi-Guizhi” for the treatment of osteoarthritis on the basis of network pharmacology and molecular docking. Comb. Chem. and High Throughput Screen. 26 (4), 743–755. 10.2174/1386207325666220512000940 35546760

[B17] Chinese Pharmacopoeia Commission (2025). Pharmacopoeia of People’s Republic of China. 2025 Edition. Beijing, China: China Medical Science Press.

[B18] Csupor D. Borcsa B. Heydel B. Hohmann J. Zupkó I. Ma Y. (2011). Comparison of a specific HPLC determination of toxic aconite alkaloids in processed Radix aconiti with a titration method of total alkaloids. Pharm. Biol. 49 (10), 1097–1101. 10.3109/13880209.2011.595011 21936629

[B19] Deng J. Han J. Chen J. Zhang Y. Huang Q. Wang Y. (2021). Comparison of analgesic activities of aconitine in different mice pain models. PLoS One 16 (4), e0249276. 10.1371/journal.pone.0249276 33793632 PMC8016268

[B20] Ding M. Wei X. Liu C. Tan X. (2024). Mahuang Fuzi Xixin decoction alleviates allergic rhinitis by inhibiting NLRP3/caspase-1/GSDMD-N-mediated pyroptosis. J. Ethnopharmacol. 327, 118041. 10.1016/J.JEP.2024.118041 38479543

[B21] Du J. Lu X. Long Z. Zhang Z. Zhu X. Yang Y. (2013). *In vitro* and *in vivo* anticancer activity of Aconitine on melanoma cell line B16. Molecules 18 (1), 757–767. 10.3390/molecules18010757 23299553 PMC6270132

[B22] Du Y. Hu J. Zhang P. Ge T. Zhou Y. (2024). Application of Sini Decoction at acupoint on gastrointestinal dysfunction in patients with sepsis: a clinical study. Med. (Baltimore) 103 (44), e40464. 10.1097/md.0000000000040464 PMC11537635 39495969

[B23] Feng W. Liu J. Zhang D. Tan Y. Cheng H. Peng C. (2021). Revealing the efficacy-toxicity relationship of Fuzi in treating rheumatoid arthritis by systems pharmacology. Sci. Rep. 11 (1), 23083. 10.1038/s41598-021-02167-5 34845218 PMC8630009

[B24] Fu Y. P. Zou Y. F. Lei F. Y. Wangensteen H. Inngjerdingen K. T. (2022). *Aconitum carmichaelii* Debeaux: a systematic review on traditional use, and the chemical structures and pharmacological properties of polysaccharides and phenolic compounds in the roots. J. Ethnopharmacol. 291, 115148. 10.1016/j.jep.2022.115148 35240238

[B25] Gao T. Bi H. Ma S. Lu J. (2010). The antitumor and immunostimulating activities of water soluble polysaccharides from Radix Aconiti, Radix Aconiti Lateralis and Radix Aconiti Kusnezoffii. Nat. Product. Commun. 5 (3), 447–455. 10.1177/1934578X1000500322 20420326

[B26] Gao Y. Fan H. Nie A. Yang K. Xing H. Gao Z. (2022). Aconitine: a review of its pharmacokinetics, pharmacology, toxicology and detoxification. J. Ethnopharmacol., 293, 115270. 10.1016/j.jep.2022.115270 35405250

[B27] Haldhar R. Raorane C. J. Bachhar V. Katin K. P. Berdimurodov E. Shazly G. A. (2025). Dual-functional Quercus palustris leaves extract as a sustainable corrosion inhibitor for low-carbon steel and its biomedical potential: electrochemical, biological, and computational insights. Sustain. Mater. Technol. 44, e01378. 10.1016/j.susmat.2025.e01378

[B28] He Y. Wei Z. Ci X. Xie Y. Yi X. Zeng Y. (2019). Effects of liquorice on pharmacokinetics of aconitine in rats. Xenobiotica 49 (12), 1485–1493. 10.1080/00498254.2019.1579007 30741588

[B29] Health Sciences Authority Government of Singapore (2025). Regulatory Overview of Chinese Proprietary Medicines. Available online at: https://www.hsa.gov.sg/chinese-proprietary-medicines/overview (Accessed March 7, 2026).

[B30] Hu Q. Liu Y. Yu J. Yang X. Yang M. He Y. (2023). The protective effect and antitumor activity of *Aconiti Lateralis Radix Praeparata* (Fuzi) polysaccharide on cyclophosphamide-induced immunosuppression in H22 tumor-bearing mice. Front. Pharmacol. 14, 1151092. 10.3389/fphar.2023.1151092 37033618 PMC10079910

[B31] Huang Q. A. Zhang Y. M. He Y. Lu J. Lin R. C. (2007). Studies on hydrolysis of aconitine. China J. Chin. Materia Medica 32 (20), 2143–2145. 18306748

[B32] Huang C. Dong J. Cheng L. Ma H. Wang F. Feng Y. (2022). Alkaloids from *Aconitum carmichaelii* alleviates DSS-induced ulcerative colitis in mice via MAPK/NF-κB/STAT3 signaling inhibition. Evid. Based Complement. Altern. Med. 2022, 6257778. 10.1155/2022/6257778 PMC9173982 35685720

[B33] Jaiswal Y. Liang Z. Yong P. Chen H. Zhao Z. (2013). A comparative study on the traditional Indian Shodhana and Chinese processing methods for aconite roots by characterization and determination of the major components. Chem. Central J. 7 (1), 169. 10.1186/1752-153X-7-169 24156713 PMC4015782

[B34] Jaiswal Y. Liang Z. Ho A. Wong L. Yong P. Chen H. (2014). Distribution of toxic alkaloids in tissues from three herbal medicine aconitum species using laser micro-dissection, UHPLC–QTOF MS and LC–MS/MS techniques. Phytochemistry 107, 155–174. 10.1016/j.phytochem.2014.07.026 25172517

[B35] Ji X. Yang M. Shen G. Or K. H. Yim W. S. Zuo Z. (2023). Safety evaluations of the processed lateral root of *Aconitum carmichaelii* Debx. And its hepatotoxicity mechanisms in rats. J. Ethnopharmacol. 301, 115801. 10.1016/j.jep.2022.115801 36216199

[B36] Jiang H. Zhang Y. Zhang Y. Wang X. Meng X. (2022). An Updated Meta-Analysis Based on the Preclinical Evidence of Mechanism of Aconitine-Induced Cardiotoxicity. Front. Pharmacol. 13, 900842. 10.3389/fphar.2022.900842 35754486 PMC9213726

[B37] Jiang C. Shen J. Wang C. Huang Y. Wang L. Yang Y. (2023). Mechanism of aconitine mediated neuronal apoptosis induced by mitochondrial calcium overload caused by MCU. Toxicol. Lett., 384, 86–95. 10.1016/j.toxlet.2023.07.014 37506855

[B38] Jiang Y. Zhang B. Lei J. Jing Y. Liu J. Yang J. (2024). Clinical application and mechanism of action of Guizhi Fuzi decoction. World Chin. Med. 19 (13), 1974–1978.

[B39] Jin X. Cheng J. Zhang Q. Ji H. Zhu C. Yang Y. (2023). Aconitine – a promising candidate for treating cold and mechanical allodynia in cancer induced bone pain. Biomed. and Pharmacother. 161, 114284. 10.1016/j.biopha.2023.114284 36868017

[B40] Joshi V. Bachhar V. Bhatia A. Rom T. Duseja M. Shukla R. K. (2025a). Green synthesis of multifunctional AgFe nanoparticles using Piper chaba extract: evaluation of antioxidant, antidiabetic, and antibacterial activities. Colloids Surfaces A Physicochem. Eng. Aspects 725, 137619. 10.1016/j.colsurfa.2025.137619

[B41] Joshi V. Bachhar V. Mishra S. S. Shukla R. K. Duseja M. (2025b). Hexagonal ZnO nanoparticles synthesised using methanolic extract of Piper chaba aerial parts for catalytic oxidation of morin, 4-nitrophenol reduction, antioxidant and antibacterial applications. J. Mol. Struct. 1348, 143550. 10.1016/j.molstruc.2025.143550

[B42] Joshi V. Bachhar V. Singh P. Duseja M. Haldhar R. Katin K. P. (2025c). Rheological and anticorrosion study of Piper chaba extract and coating for mild steel in 2M H2SO4. Colloids Surfaces A Physicochem. Eng. Aspects, 708, 135989. 10.1016/j.colsurfa.2024.135989

[B43] Joshi V. Mishra S. S. Haldhar R. Bachhar V. Duseja M. Kim S.-C. (2026). Sustainable valorisation of onion peel waste through silver nanoparticle synthesis: antibacterial, antidiabetic and computational insights. J. Mol. Struct., 1352, 144570. 10.1016/j.molstruc.2025.144570

[B44] Kimura I. Makino M. Takamura Y. M. Islam A. Kimura M. (1994). Positive chronotropic and inotropic effects of higenamine and its enhancing action on the aconitine-induced tachyarrhythmia in isolated Murine Atria. Jpn. J. Pharmacol. 66 (1), 75–80. 10.1254/jjp.66.75 7861670

[B45] Lau C. M. Azman M. (2021). The roots of WOE. Malays. J. Emerg. Med. 5 (2), 213.

[B46] Li H. Liu L. Zhu S. Liu Q. (2016). Case reports of aconite poisoning in mainland China from 2004 to 2015: a retrospective analysis. J. Forensic Leg. Med., 42, 68–73. 10.1016/j.jflm.2016.05.016 27266651

[B47] Li X. Gu L. Yang L. Zhang D. Shen J. (2017). Aconitine: a potential novel treatment for systemic lupus erythematosus. J. Pharmacol. Sci. 133 (3), 115–121. 10.1016/j.jphs.2017.01.007 28302448

[B48] Li F. Guo C. Zhang S. Zheng B. Sun K. Shi J. (2023). Exploring the role and mechanism of Fuzi decoction in the treatment of osteoporosis by integrating network pharmacology and experimental verification. J. Orthopaedic Surg. Res. 18 (1), 508. 10.1186/s13018-023-03842-1 PMC10354906 37464262

[B49] Li X.-Y. Zhou L. Jiang Z.-H. Liang Y. Fan Y.-G. Ding Z.-H. (2025). Integrated toxicology of *Aconitum carmichaelii* Debx.: bridging traditional toxicity-efficacy understanding and modern toxicology. Front. Pharmacol., 16. 10.3389/fphar.2025.1667059 PMC12620505 41256257

[B50] Liang X. Su W. Zhang W. Wang S. Wu X. Li X. (2025). An overview of the research progress on *Aconitum carmichaelii* Debx.:active compounds, pharmacology, toxicity, detoxification, and applications. J. Ethnopharmacol., 337, 118832. 10.1016/j.jep.2024.118832 39306209

[B51] Liao L. Z. Chen Y. L. Lu L. H. Zhao Y. H. Guo H. L. Wu W. K. (2013). Polysaccharide from Fuzi likely protects against starvation-induced cytotoxicity in H9c2 cells by increasing autophagy through activation of the AMPK/mTOR pathway. Am. J. Chin. Med. 41 (2), 353–367. 10.1142/S0192415X13500262 23548125

[B52] Lina Z. Xiu L. Xin Z. Wenjuan L. I. Ye Z. (2025). Study on the mechanism of Fuzi Lizhong decoction in the treatment of colorectal cancer of spleen kidney deficiency from the perspective of intestinal flora and hypoxia inducible factor-1α signalling pathway. J. Tradit. Chin. Med. 45 (4), 845–851. 10.19852/j.cnki.jtcm.2025.04.013 40810230 PMC12340584

[B53] Liu F. Yu X. H. Li F. Tan Y. Y. Qiao Y. J. (2006). Determination of three kind of diester diterpenoid alkaloids (DDAs) in Aconitum carmichaeli and its processed products by HPLC. Zhongguo Zhong Yao Za Zhi 31 (14), 1160–1162. 17048584

[B54] Liu X. X. Jian X. X. Cai X. F. Chao R. B. Chen Q. H. Chen D. L. (2012). Cardioactive C_19_-diterpenoid alkaloids from the lateral roots of Aconitum carmichaeli “Fu Zi”. Chem. Pharm. Bull. (Tokyo) 60 (1), 144–149. 10.1248/cpb.60.144 22223386

[B55] Liu M. Cao Y. Lv D. Zhang W. Zhu Z. Zhang H. (2017). Effect of processing on the alkaloids in Aconitum tubers by HPLC-TOF/MS. J. Pharm. Analysis 7 (3), 170–175. 10.1016/j.jpha.2017.01.001 29404034 PMC5790648

[B56] Liu B. Cao Y. Wang D. Zhou Y. Zhang P. Wu J. (2021). Zhen-Wu-Tang induced mitophagy to protect mitochondrial function in chronic glomerulonephritis via PI3K/AKT/mTOR and AMPK pathways. Front. Pharmacol. 12, 777670. 10.3389/fphar.2021.777670 35757387 PMC9231558

[B57] Liu J. Zhang D. Zhou Y. Wu J. Feng W. Peng C. (2025). Fuzi alleviates cold-related rheumatoid arthritis via regulating gut microbiota and microbial bile acid metabolism. Chin. Med. 20 (1), 64. 10.1186/s13020-025-01123-z 40375326 PMC12079872

[B58] Lu X. Zhang L. Li P. Wang J. Li R. Huang Y. (2017). The protective effects of compatibility of aconiti lateralis radix praeparata and zingiberis rhizoma on rats with heart failure by enhancing mitochondrial biogenesis via Sirt1/PGC-1α pathway. Biomed. Pharmacother. 92, 651–660. 10.1016/j.biopha.2017.05.117 28578259

[B59] Lv Y. Bo H. Yang L. Li X. Li F. (2011). Comparasion of polysaccharides in parent root, daughter root and rootlet of Aconitum carmichaeli. China J. Chin. Materia Medica 36 (9), 1154–1157. 10.4268/cjcmm20110909 21842639

[B60] Ministry Health and Welfare (2022). Taiwan Herbal Pharmacopeia. 4th edition. Ministry of Health and Welfare. Taipei, Taiwan:

[B61] Ministry of Food and Drug Safety (2015). Regulation on Approval and Notification of Herbal (Crude) Medicinal Preparations etc. Available online at: https://www.mfds.go.kr/eng/brd/m_27/view.do?seq=70932 (Accessed March 7, 2026).

[B62] Ministry of Food and Drug Safety (2020). The Korean Pharmacopoeia. 12th edition. Cheongju-si, Republic of Korea: Ministry of Food and Drug Safety.

[B63] Ministry of Health and Family Welfare (2016). The Drugs and Cosmetics Rules, 1945. Available online at: https://cdsco.gov.in/opencms/export/sites/CDSCO_WEB/Pdf-documents/acts_rules/2016DrugsandCosmeticsAct1940Rules1945.pdf (Accessed March 7, 2026).

[B64] Mizugaki M. Ito K. Ohyama Y. Konishi Y. Tanaka S. Kurasawa K. (1998). Quantitative analysis of *aconitum* alkaloids in the urine and serum of a Male attempting suicide by oral intake of aconite extract. J. Anal. Toxicol. 22 (4), 336–340. 10.1093/jat/22.4.336 9681338

[B65] Muroi M. Kimura I. Kimura M. (1990). Blocking effects of hypaconitine and aconitine on nerve action potentials in phrenic nerve-diaphragm muscles of mice. Neuropharmacol. 29(6), 567–572. 10.1016/0028-3908(90)90069-4 2385329

[B66] National Pharmaceutical Regulatory Agency (2016).Traditional Product Li Chung Pill (Ubat bebola) Found to Contain Toxic Alkaloids. Available online at: https://www.npra.gov.my/index.php/my/component/content/article/39-english/press-release/press-release-2016/991-kenyataan-akhbar-produk-tradisional-li-chung-pill-ubat-bebola-dikesan-mengandungi-alkaloid-bertoksik.html?Itemid=1392 (Accessed March 7, 2026).

[B67] Nyirimigabo E. Xu Y. Li Y. Wang Y. Agyemang K. Zhang Y. (2015). A review on phytochemistry, pharmacology and toxicology studies of *Aconitum* . J. Pharm. Pharmacol. 67 (1), 1–19. 10.1111/jphp.12310 25244533

[B68] Page M. J. McKenzie J. E. Bossuyt P. M. Boutron I. Hoffmann T. C. Mulrow C. D. (2021). The PRISMA 2020 statement: an updated guideline for reporting systematic reviews. BMJ 372, n71. 10.1136/bmj.n71 33782057 PMC8005924

[B69] Pang M. Song X. Miao Y. Wang Y. Zhou C. Geng Z. (2023). *Aconitum carmichaelii* triggers neurotoxicity and Parkinson-like symptoms through initiation of ROS-mitochondrial apoptosis and the Nox5/DJ-1 signaling pathway. Biomed. Eng. Mater. 1 (2), e12019. 10.1002/bmm2.12019

[B70] Peng C. Zheng T. Yang F. Li Y. X. Zhang D. K. (2009). Study of neurotoxic effects and underlying mechanisms of aconitine on cerebral cortex neuron cells. Archives Pharmacal Res. 32 (11), 1533–1543. 10.1007/s12272-009-2105-1 20091266

[B71] Peng F. Zhang N. Wang C. Wang X. Huang W. Peng C. (2020). Aconitine induces cardiomyocyte damage by mitigating BNIP3-dependent mitophagy and the TNFα-NLRP3 signalling axis. Cell Prolif. 53 (1), e12701. 10.1111/cpr.12701 31657084 PMC6985658

[B72] Pharmaceutical and Medical Device Regulatory Science Society of Japan (2021). The Japanese Pharmacopoeia. 18th edition. Tokyo, Japan: Yakuji Nippo Ltd.

[B73] Plants of the World Online (2026). Aconitum carmichaelii Debeaux. Kew. London, United Kingdom: Royal Botanic Gardens. Available online at: https://powo.science.kew.org/ (Accessed March 7, 2026).

[B74] Sheth S. Tan E. C. Tan H. H. Tay L. (2015). Herb-induced cardiotoxicity from accidental aconitine overdose. Singap. Med. J. 56 (7), e116–e119. 10.11622/smedj.2015114 26243980 PMC4520923

[B75] Shu X. Hou D. (2008). Comparative study on polysaccharide content of Aconite at different harvesting stages. Chin. Tradit. Pat. Med. 30, 1512–1514. 10.3969/j.issn.1001-1528.2008.10.033

[B76] Shu X. Zhao X. Hou D. (2009). Study on quality changes of Radix Aconiti Lateralis Praeparata. J. Chin. Med. Mater. 32 (1), 29–31. 10.13863/j.issn1001-4454.2009.01.051 19445117

[B77] Singhuber J. Zhu M. Prinz S. Kopp B. (2009). *Aconitum* in Traditional Chinese medicine—a valuable drug or an unpredictable risk? J. Ethnopharmacol. 126(1), 18–30. 10.1016/j.jep.2009.07.031 19651200

[B78] Song J. Z. Han Q. B. Qiao C. F. But P. P. H. Xu H. X. (2010). Development and validation of a rapid capillary zone electrophoresis method for the determination of aconite alkaloids in aconite roots. Phytochem. Anal. 21 (2), 137–143. 10.1002/pca.1168 19810124

[B79] Song W. T. Cheng F. F. Xu L. Lin C. R. Liu J. X. (2012). Chinese medicine shenfu injection for heart failure: a systematic review and meta-analysis. Evidence-Based Complement. Altern. Med. 2012 (1), 713149. 10.1155/2012/713149 PMC3348640 22611430

[B80] Sun G. B. Sun H. Meng X. B. Hu J. Zhang Q. Liu B. (2014). Aconitine-induced Ca2+ overload causes arrhythmia and triggers apoptosis through p38 MAPK signaling pathway in rats. Toxicol. Appl. Pharmacol. 279(1), 8–22. 10.1016/j.taap.2014.05.005 24840785

[B81] Tai C. J. El-Shazly M. Wu T. Y. Lee K. T. Csupor D. Hohmann J. (2015). Clinical aspects of aconitum preparations. Planta Medica 81 (12-13), 1017–1028. 10.1055/s-0035-1546183 26166138

[B82] Tao L. Shen S. Li X. (2013). Future prospects of Qiliqiangxin on heart failure: epigenetic regulation of regeneration. Front. Genet. 4, 221. 10.3389/fgene.2013.00221 24167520 PMC3807038

[B83] Tong P. Wu C. Wang X. Hu H. Jin H. Li C. (2013). Development and assessment of a complete-detoxication strategy for Fuzi (lateral root of *Aconitum carmichaeli*) and its application in rheumatoid arthritis therapy. J. Ethnopharmacol. 146 (2), 562–571. 10.1016/j.jep.2013.01.025 23376046

[B84] Wada K. Nihira M. Hayakawa H. Tomita Y. Hayashida M. Ohno Y. (2005). Effects of long-term administrations of aconitine on electrocardiogram and tissue concentrations of aconitine and its metabolites in mice. Forensic Sci. Int. 148(1), 21–29. 10.1016/j.forsciint.2004.04.016 15607586

[B85] Wang Y. Wang S. Liu Y. Yan L. Dou G. Gao Y. (2006). Characterization of metabolites and cytochrome P450 isoforms involved in the microsomal metabolism of aconitine. J. Chromatogr. B 844 (2), 292–300. 10.1016/j.jchromb.2006.07.059 16949890

[B86] Wang Z. Wen J. Xing J. He Y. (2006). Quantitative determination of diterpenoid alkaloids in four species of *Aconitum* by HPLC. J. Pharm. Biomed. Analysis 40 (4), 1031–1034. 10.1016/j.jpba.2005.08.012 16181762

[B87] Wang Y. Zhang X. Li J. Zhang Y. Guo Y. Chang Q. (2021). Sini decoction ameliorates colorectal cancer and modulates the composition of gut microbiota in mice. Front. Pharmacol. 12, 609992. 10.3389/fphar.2021.609992 33776762 PMC7991589

[B88] Wang Y. J. Tao P. Wang Y. (2021). Attenuated structural transformation of Aconitine during sand frying process and antiarrhythmic effect of its converted products. Evid.-Based Complement. Altern. Med., 2021(1), 7243052. 10.1155/2021/7243052 PMC8560236 34733344

[B89] Wang H. Liu Y. Guo Z. Wu K. Zhang Y. Tian Y. (2022). Aconitine induces cell apoptosis via mitochondria and death receptor signaling pathways in hippocampus cell line. Res. Veterinary Sci. 143, 124–133. 10.1016/j.rvsc.2022.01.001 35026629

[B90] Wei X. Ding M. Liang X. Zhang B. Tan X. Zheng Z. (2023). Mahuang Fuzi Xixin decoction ameliorates allergic rhinitis and repairs the airway epithelial barrier by modulating the lung microbiota dysbiosis [Original Research]. Front. Microbiol. 14, 1206454. 10.3389/fmicb.2023.1206454 37645224 PMC10461068

[B91] Wen R. Q. Li D. H. Zhao X. Wang J. B. Zhao Y. L. Zhang P. (2013). Rationality of the processing methods of aconiti lateralis radix (Fuzi) based on chemical analysis. Yao Xue Xue Bao 48 (2), 286–290. 23672028

[B92] Wu J. Wang X. Chung Y. Y. Koh C. H. Liu Z. Guo H. (2017). L-Type calcium channel inhibition contributes to the proarrhythmic effects of Aconitine in human cardiomyocytes. PLoS One 12 (1), e0168435. 10.1371/journal.pone.0168435 28056022 PMC5215924

[B93] Wu J. J. Guo Z. Z. Zhu Y. F. Huang Z. J. Gong X. Li Y. H. (2018). A systematic review of pharmacokinetic studies on herbal drug Fuzi: implications for Fuzi as personalized medicine. Phytomedicine, 44, 187–203. 10.1016/j.phymed.2018.03.001 29526584

[B94] Xiang G. Xing N. Wang S. Zhang Y. (2023). Antitumor effects and potential mechanisms of aconitine based on preclinical studies: an updated systematic review and meta-analysis. Front. Pharmacol. 14, 1172939. 10.3389/fphar.2023.1172939 37180714 PMC10174313

[B95] Xing X. Guo J. Mo J. Li H. Zhang H. Shao B. (2023). Qili Qiangxin capsules for chronic heart failure: A GRADE-assessed clinical evidence and preclinical mechanism. Front. Cardiovasc. Med. 9, 1090616. 10.3389/fcvm.2022.1090616 36712277 PMC9873992

[B96] Xu F. F. Xie X. F. Hu H. Y. Tong R. S. Peng C. (2024). Shenfu injection: a review of pharmacological effects on cardiovascular diseases. Front. Pharmacol. 15, 1279584. 10.3389/fphar.2024.1279584 38420190 PMC10899515

[B97] Yan P. Mao W. Jin L. Fang M. Liu X. Lang J. (2020). Crude Radix Aconiti Lateralis preparata (Fuzi) with *Glycyrrhiza* reduces inflammation and ventricular remodeling in mice through the TLR4/NF-κB pathway. Mediat. Inflamm. 2020, 5270508. 10.1155/2020/5270508 33132755 PMC7593747

[B98] Yang H. Liu L. Gao W. Liu K. Qi L. W. Li P. (2014). Direct and comprehensive analysis of ginsenosides and diterpene alkaloids in Shenfu injection by combinatory liquid chromatography–mass spectrometric techniques. J. Pharm. Biomed. Anal. 92, 13–21. 10.1016/j.jpba.2013.12.041 24469096

[B99] Yang L. Wang Y. Huang G. Li J. Zhang Z. Ma Z. (2018). Simultaneous evaluation of the influence of *Panax ginseng* on the pharmacokinetics of three diester alkaloids after oral administration of Aconiti Lateralis Radix in rats using UHPLC/QQQ-MS/MS. Evid. Based Complement. Altern. Med. 2018, 6527549. 10.1155/2018/6527549 30622607 PMC6304572

[B100] Yang M. Ji X. Zuo Z. (2018). Relationships between the toxicities of Radix Aconiti Lateralis Preparata (Fuzi) and the toxicokinetics of its main diester-diterpenoid alkaloids. Toxins (Basel) 10 (10), 391. 10.3390/toxins10100391 30261585 PMC6215299

[B101] Yang L. Chen Y. Zhou J. Sun J. Jiang W. Liu T. (2021). Aconitine induces mitochondrial energy metabolism dysfunction through inhibition of AMPK signaling and interference with mitochondrial dynamics in SH-SY5Y cells. Toxicol. Lett. 347, 36–44. 10.1016/j.toxlet.2021.04.020 33945864

[B102] Yang J. Zhang Y. Yi H. Liao Y. Shu L. Zhang S. (2022). Fuzi-Lizhong decoction alleviates nonalcoholic Fatty liver disease by blocking TLR4/MyD88/TRAF6 signaling. Evidence-Based Complementary Altern. Med. 2022 (1), 1637701–1637710. 10.1155/2022/1637701 PMC9440633 36065267

[B103] Yin T. Zhou H. Cai L. Ding Z. (2019). Non-alkaloidal constituents from the genus *Aconitum*: a review [10.1039/C9RA01219B]. RSC Adv. 9 (18), 10184–10194. 10.1039/C9RA01219B 35520886 PMC9062526

[B104] Yue C. (2015). The comparative studies on the quality of the main cultivation area of aconite. Master’s thesis. Mianyang, China: Southwest University of Science and Technology. Available online at: https://d.wanfangdata.com.cn/thesis/ChJUaGVzaXNOZXdTMjAyMTEyMDESB0Q2NTE3NjUaCHZ6cnBoM3c1.

[B105] Zhang M. Peng C. S. Li X. B. (2017). Human intestine and liver microsomal metabolic differences between C19-diester and monoester diterpenoid alkaloids from the roots of *Aconitum carmichaelii* debx. Toxicol. Vitro 45, 318–333. 10.1016/j.tiv.2017.09.011 28919359

[B106] Zhang Y. Zong X. Wu J. L. Liu Y. Liu Z. Zhou H. (2020). Pharmacokinetics and tissue distribution of eighteen major alkaloids of *Aconitum carmichaelii* in rats by UHPLC-QQQ-MS. J. Pharm. Biomed. Analysis 185, 113226. 10.1016/j.jpba.2020.113226 32163851

[B107] Zhang J. Li D. Zhong D. Zhou Q. Yin Y. Gao J. (2022). Processed lateral root of *Aconitum carmichaelii* Debx.: a review of cardiotonic effects and cardiotoxicity on molecular mechanisms. Front. Pharmacol. 13, 1026219. 10.3389/fphar.2022.1026219 36324672 PMC9618827

[B108] Zhang Z. Zhou W. Zhang Y. Hu Y. Mou J. Wang X. (2024). Advances for anti-inflammatory ingredients and activities of Fuzi (lateral root of *Aconitum carmichaelii* Debx.). Int. J. Food Prop. 27 (1), 53–70. 10.1080/10942912.2023.2293461

[B109] Zhao X. Hou D. Shu X. (2009). Effects of plant hormones on the determination of polysaccharide of *Aconitum carmichaelii* . Lishizhen Med. Materia Medica Res. 20 (9), 2195–2196. 10.3969/j.issn.1008-0805.2009.09.042

[B110] Zhao X. S. Wang H. Xu Y. Hou D. B. (2009). Content determination of polysaccharide in aconitum carmichaili of Jiangyou. Anhui Agric. Sci. 37 (2), 650–651. 10.3969/j.issn.0517-6611.2009.02.093

[B111] Zhao Y. Bu Q. Zhou Y. Lv L. Yan G. Chen B. (2010). Mechanism study of Aconitum-induced neurotoxicity in PC12 cells: involvement of dopamine release and oxidative damage. NeuroToxicology, 31(6), 752–757. 10.1016/j.neuro.2010.06.005 20600291

[B112] Zhao D. Wang J. Cui Y. Wu X. (2012). Pharmacological effects of Chinese herb aconite (Fuzi) on cardiovascular system. J. Traditional Chin. Med. 32(3), 308–313. 10.1016/S0254-6272(13)60030-8 23297548

[B113] Zhao P. Tian Y. Geng Y. Zeng C. Ma X. Kang J. (2024). Aconitine and its derivatives: bioactivities, structure-activity relationships and preliminary molecular mechanisms. Front. Chem. 12, 1339364. 10.3389/fchem.2024.1339364 38318112 PMC10839071

[B114] Zhen Z. Xia L. You H. Jingwei Z. Shasha Y. Xinyi W. (2021). An integrated gut microbiota and network pharmacology study on Fuzi-Lizhong pill for treating diarrhea-predominant irritable bowel syndrome. Front. Pharmacol. 12, 746923. 10.3389/fphar.2021.746923 34916934 PMC8670173

[B115] Zheng M. Hu Z. Wang Y. Wang C. Zhong C. Cui W. (2023). Zhen Wu decoction represses renal fibrosis by invigorating tubular NRF2 and TFAM to fuel mitochondrial bioenergetics. Phytomedicine 108, 154495. 10.1016/j.phymed.2022.154495 36257219

[B116] Zhou Y. H. Piao X. M. Liu X. Liang H. H. Wang L. M. Xiong X. H. (2013). Arrhythmogenesis Toxicity of Aconitine Is Related to Intracellular Ca^2+^ Signals [Research Paper]. Int. J. Med. Sci. 10 (9), 1242–1249. 10.7150/ijms.6541 23935402 PMC3739024

[B117] Zhou G. Tang L. Zhou X. Wang T. Kou Z. Wang Z. (2015). A review on phytochemistry and pharmacological activities of the processed lateral root of *Aconitum carmichaelii* Debeaux. J. Ethnopharmacol. 160, 173–193. 10.1016/j.jep.2014.11.043 25479152

[B118] Zhou J. C. Jin C. C. Wei X. L. Xu R. B. Wang R. Y. Zhang Z. M. (2023). Mesaconine alleviates doxorubicin-triggered cardiotoxicity and heart failure by activating PINK1-dependent cardiac mitophagy [Original Research]. Front. Pharmacol. 14, 1118017. 10.3389/fphar.2023.1118017 37124193 PMC10132857

[B119] Zong X. Yan X. Wu J. L. Liu Z. Zhou H. Li N. (2019). Potentially cardiotoxic diterpenoid alkaloids from the roots of *Aconitum carmichaelii* . J. Nat. Prod. 82 (4), 980–989. 10.1021/acs.jnatprod.8b01039 30892884

